# Conjoint analysis of physio-biochemical, transcriptomic, and metabolomic reveals the response characteristics of *solanum nigrum* L. to cadmium stress

**DOI:** 10.1186/s12870-024-05278-z

**Published:** 2024-06-17

**Authors:** Juncai Wang, Xunfeng Chen, Shaohua Chu, Kashif Hayat, Yaowei Chi, Xiaofeng Liao, Hongliang Zhang, Yuangui Xie, Pei Zhou, Dan Zhang

**Affiliations:** 1 Guizhou Academy of Sciences, Guiyang, Guizhou, 550001 China; 2 Biofuels Institute, School of Environment and Safety Engineering, Jiangsu University, Zhenjiang, 212013 China; 3 School of Agriculture and Biology, Shanghai Jiao Tong University, Shanghai, 200240 China; 4 The Land Greening Remediation Engineering Research Center of Guizhou Province, Guiyang, 550001 China; 5 Key Laboratory of Pollution Exposure and Health Intervention, Interdisciplinary Research Academy, Zhejiang Shuren University, Hangzhou, 310015 China; 6 Guizhou University, Guiyang, 550025 China

**Keywords:** Cadmium toxicity, *solanum nigrum* L., Metabolomics analysis, Transcriptomics analysis, Plant physiology

## Abstract

Cadmium (Cd) is a nonessential element in plants and has adverse effects on the growth and development of plants. However, the molecular mechanisms of Cd phytotoxicity, tolerance and accumulation in hyperaccumulators *Solanum nigrum* L. has not been well understood. Here, physiology, transcriptome, and metabolome analyses were conducted to investigate the influence on the *S. nigrum* under 0, 25, 50, 75 and 100 µM Cd concentrations for 7 days. Pot experiments demonstrated that compared with the control, Cd treatment significantly inhibited the biomass, promoted the Cd accumulation and translocation, and disturbed the balance of mineral nutrient metabolism in *S. nigrum*, particularly at 100 µM Cd level. Moreover, the photosynthetic pigments contents were severely decreased, while the content of total protein, proline, malondialdehyde (MDA), H_2_O_2_, and antioxidant enzyme activities generally increased first and then slightly declined with increasing Cd concentrations, in both leaves and roots. Furthermore, combined with the previous transcriptomic data, numerous crucial coding-genes related to mineral nutrients and Cd ion transport, and the antioxidant enzymes biosynthesis were identified, and their expression pattern was regulated under different Cd stress. Simultaneously, metabolomic analyses revealed that Cd treatment significantly changed the expression level of many metabolites related to amino acid, lipid, carbohydrate, and nucleotide metabolism. Metabolic pathway analysis also showed that *S. nigrum* roots activated some differentially expressed metabolites (DEMs) involved in energy metabolism, which may enhance the energy supply for detoxification. Importantly, central common metabolism pathways of DEGs and DEMs, including the “TCA cycle”, “glutathione metabolic pathway” and “glyoxylate and dicarboxylate metabolism” were screened using conjoint transcriptomics and metabolomics analysis. Our results provide some novel evidences on the physiological and molecular mechanisms of Cd tolerance in hyperaccumulator *S. nigrum* plants.

**Supplementary Information:**

The online version contains supplementary material available at 10.1186/s12870-024-05278-z.

## Introduction

Over recent decades, with the intensification of anthropogenic and industrial activities such as sewage irrigation, mining, smelters, waste incineration and the overuse of phosphate fertilizer, heavy metals are released into the environmental matrices, including soil [1]. Among different heavy metals, cadmium (Cd) is hazardous and poses an unprecedented threat to crop quality and yield, living organism health and environmental quality even at low concentrations [2, 3]. As a nonessential element, Cd may be absorbed by plant roots and subsequently translocated and accumulated in different organs, and has negative physio-biochemical effects on plants [4, 5]. The effects of Cd on plants are complicated and depend on plant species, the exposure time and intensity of Cd, and the type of plant tissue [5, 6]. Therefore, exploration of the response changes of Cd in plants is deemed fundamental.

In plants, Cd imposes toxicity by over generating reactive oxygen species (ROS, e.g. OH^−^, H_2_O_2_, O_2_^−^ and ^1^O_2_), triggering oxidative stress that disrupts redox homeostasis [5–7]. This redox imbalance results in accelerated lipid peroxidation changing plasma membrane fluidity and permeability, and degrading functional biomolecules (e.g., chlorophylls, proteins, RNA, DNA), so plants need to maintain a balance between scavenging and ROS generation [7, 8]. To alleviate oxidative stress, plants usually adopt various defense systems, includes activation of efficient antioxidant defense system [9–11]. For instance, plants usually produce various antioxidant enzymes, superoxide dismutase (SOD), peroxidase (POD), catalase (CAT), and ascorbate peroxidase (APX), which can alleviate the ROS toxicity [6, 12]. The mulberry can efficaciously reduce oxidative stress via enhancing the POD, CAT and APX activities under different Cd concentrations [11]. Similarly, the CAT and SOD activities of Cd treatment *Populus* × *canadensis* ‘Neva’ leaves were significantly increased compared with control treatment [13]. Conversely, Labidi et al. [14] found that the activity of CAT and SOD in *Cucurbita pepo* decreased under Cd stress. In addition, proline and soluble proteins are also crucial osmotic regulatory substances that resist the unbalance of oxidative stress caused by Cd stress [15]. Numerous studies have confirmed that excessive Cd significantly increased the content of proline and soluble protein in plants [16–18]. At present, changes in the antioxidative system and osmotic substances in plants under Cd stress have been extensively studied by earlier researchers [6, 8]. However, the research results are inconsistent due to differences in plant species, plant organs, Cd stress time and stress concentrations. Thus, it is imperative for us to further investigate the response of the plant antioxidant enzyme system and osmotic substances to Cd stress, especially on the expression level of antioxidant system related genes involved in ROS scavenging.

To date, numerous studies have demonstrated that plants need a whole range of 16 mineral elements, such as macro-nutrients elements are nitrogen (N), phosphorus (P), potassium (K), calcium (Ca), trace elements iron (Fe), zinc (Zn), copper (Cu), boron (B), and sodium (Na), etc [19]. These mineral nutrients have been confirmed to be closely related to regulating the uptake, translocation, accumulation and physiological metabolism processes of toxicity elements in plants [19, 20]. Some recent studies have focused on the interaction between mineral nutrient metabolism processes and Cd phytotoxicity, as well as the influence of this interaction on Cd absorption, translocation, accumulation and detoxification [20–22]. For instance, Zhang et al. [23] found that the content of N, Ca, Mg, K and P was significantly reduced both in roots and shoots of *Medicago sativa* in Cd polluted soil. Cd detoxification in ryegrass plants can be facilitated by exogenous P addition [24]. Additionally, recent studies have found that some mineral elements, for example Ca, Fe, Mn and Cu could alleviate Cd toxicity for many plants [18, 22, 25]. Nevertheless, the limited information is available about the interaction between mineral elements and Cd, and the potential molecular toxicity mechanism in plants remains elusive.

In recent years, omics techniques, for instance transcriptome and metabolome have attracted increasing attention from researchers owing to their ability to provide abundant detailed and global molecular mechanistic information on plant abiotic stress [26, 27]. These techniques have been utilized to explore the transcriptome expression profiles and metabolomics regulatory networks in several plant response to the Cd environment, for instance *Arabidopsis halleri* [28], *Sedum plumbizincicola* [29], *Oryza sativa* L [30]. *Solanum nigrum* L [31, 32]. For instance, Li et al. [13] found that numerous genes are involved in the antioxidant defense system, transduction and transporters, and they synergistically regulate the molecular mechanism of *Populus* × *canadensis* under Cd stress by transcriptomic analysis. It has been documented that the metabolites involved in antioxidant enzyme system and osmotic balance varied remarkably in the *Amaranthus hypochondriacus* under Cd stress using LC-MS metabolomics [33]. Furthermore, a multi-omics analysis revealed the vital roles of vacuolar sequestration and the pectin of cell wall fixation in *Brassica napus* response to Cd tolerance [34]. Similarly, combined transcriptome and metabolome analysis elucidate pivotal metabolic pathways responses to Cd stress in *Ipomoea batatas* L [21]. Therefore, omics analysis, especially conjoint multi-omics analysis can offer a better understanding physio-biochemical and molecular reactions of plants subjected to abiotic stress, which will probably be better to unravel the influential and regulatory mechanisms of adverse environmental factors in plants.

On the basis of previous studies, *Solanum nigrum* L. has shown great detoxification and tolerance to Cd contaminated soil and was chosen as the materials for this study [35–39]. We hypothesized that Cd stress would perturb the dynamic balance of morphophysiological properties such as biomass, osmosis substances, antioxidant enzyme systems and mineral element absorption by inducing the expression levels of relevant genes/metabolites in *S. nigrum*. The main focuses of the present study were: (a) to investigate the physiological changes of *S. nigrum* in response to Cd stress; (b) to identify elite resources of genes/metabolites and metabolism pathways possibly contributing to resistance to Cd toxicity. The current research will deepen the understanding of the effect mechanisms underlying Cd translocation and accumulation in *S. nigrum*, as well as ultimately contribute to new insights regarding improved efficiency of phytoremediation and heavy metal tolerance.

## Materials and methods

### Plant materials preparation and culture

The *S. nigrum* seeds were purchased from Shouguang Youhe Agricultural Technology Co, Ltd. (Shandong, China). As described previously [37], the seeds were surface-sterilized and soaked in distilled water for 10 h. Subsequently, the seeds were sown into plastic pots filled with substrate soil and germinated in growth chamber (25℃, 16 h light/8 h dark and 70% relative humidity) [17, 36, 37]. After emergence of 5–7 true leaves, the seedlings were transferred and precultured to a hydroponic basin (55 × 35 × 25 cm). Based on our previous experiment results, each treated pots contained six seedlings using half strength Hoagland nutrient solution, and full-strength Hoagland solution for 10 days, respectively [37]. The standard pH (6.5 ± 0.1) of the basic solution was maintained using NaOH/HCl.

### Pot experiments

An experiment in a pot was conducted using a completely randomized design (CRD) in a greenhouse after 20 d of preculture, according to the method of Wang et al. [32, 37]. The seedlings were exposed to five Cd concentrations treatments (supplied as CdCl_2_‧2.5 H_2_O), i.e., CK (0 µM Cd + HS only), 25, 50, 75 and 100 µM for 7 days described by Wang et al. (2021b). Each treatment was replicated 3 times in six basins, and each basin contained nine plants. The nutrient solution was replaced every 3 d. The plant culture conditions were consistent with our previous study (a 16/8 h light/dark photoperiod, a temperature of 18 ~ 25℃ and a relative air humidity of 65 ~ 75%) [37]. After 7 days, the seedlings were carefully harvested, washed and collected according to our previous study [37].

### Analysis of plant morphological parameters

To estimate plant growth parameters, all fresh seedlings samples were divided into roots, stems and leaves. The root and shoot lengths were determined using measuring scale. The dry weight of plant tissues (roots, stems and leaves) was measured by an electronic weighing balance. All samples were dried at 105 °C for 10 min in an oven, and dried at 75 °C until reaching invariable weight, and then weighed [35, 36].

### Analysis of cd and mineral content

The oven-dried samples were ground, sieved, and then digested using the mixed acid solution digestion (HNO_3_: HClO_4_, v: v = 4:1). The Cd and mineral elements (K, Ca, Mg, Fe, Cu, Zn, Mn) contents in digested solutions were measured [22]. The content of N was determined by the Kjeldahl method, and P contents was examined by Mo-Sb colorimetry with a Continuous Flow Analyzer (Flowsys; Systea, Anagni, Italy) [40].

To further evaluate the Cd and mineral uptake capacity of plants, the translocation factor (TF) of each element in *S. nigrum* was calculated by the following equations [35]:

$$\text{TF}\text{=}\frac{\text{The element content in plant aboveground parts (stem and leaf)}}{\text{The element content in plant roots }}$$

### Determination of photosynthetic pigments, lipid peroxidation, antioxidant enzyme activities, proline and H_2_O_2_

The photosynthetic pigment was extracted from fresh leaves (0.3 g) by using 80% acetone in darkness, and the absorbance of the extracted solution was determined according to Lichtenthaler [41]. The content of photosynthetic pigments (chlorophyll a, Chl a, chlorophyll b, Chl b, and carotenoids, Car) was calculated at 470 nm, 645 nm, and 663 nm.

To estimate the content of lipid peroxidation, antioxidant enzyme activities, proline and H_2_O_2_ in leaves and root, the fresh roots and leaves samples (0.3 g) were ground and homogenized in a mortar with 4.5 mL precooled 0.9% (w/v) phosphate buffer solution (0.1 mol L^− 1^, pH = 7.4). After centrifugation at 12 000 rpm for 20 min at 4 ℃, and the supernatant was collected for further testing. The lipid peroxidation content was measured by determining malondialdehyde (MDA) content using an assay kit (Code No. A003-3-1). Four antioxidant enzymatic activities (POD, CAT, SOD and APX) were also determined by using a series of reagent kits, including POD (Code No. A084-3-1), CAT (Code No. A007-1-1), SOD (Code No. A001-1-1), and APX (Code No. A123-1-1). The contents of total protein (TP), proline (Pro) and H_2_O_2_ in the leaves and roots were measured using assay kits, including TP (Code No. A045-2-1), Pro (Code No. A107-1-1) and H_2_O_2_ (Code No. A064-1-1) [35, 36, 42]. All parameters were measured as described in the manufacturer’s protocols, and all reagent kits were obtained from Nanjing Jiancheng Bioengineering Institute, China.

### Multiomics data

Our previously published metabolomics and transcriptome data for *S. nigrum* exposed to five different Cd concentrations for 7 d [32]. This study used the same experiment and unpublished data to reveal the Cd toxic mechanism to *S. nigrum* from physiological (mineral uptake, antioxidant enzyme activities, Cd transport, etc.) and key metabolism pathways. In addition, in this study, 14 candidates differentially expressed genes (DEGs, 5 downregulated and 9 upregulated) were screened for further quantitative real-time PCR analysis. Detail information of sequencing data is described in the Supporting Information (Text S1) and our previous research [32]. The primers for all genes are listed in Table S1.

### Statistics analysis

Date were statistically analyzed using a one-way analysis of variance (ANOVA) followed by Tukey’s test (*P*<0.05) with IBM SPSS software 24.0 (Chicago, USA). All values are the mean ± standard deviation per treatment (*n* = 3). All diagrams were drawn using OriginPro 2021 and Genescloud (http://www.genescloud.cn/home) [43]. Principal component analysis (PCA) was utilized to examine the intrinsic variation in the physiological parameters and to reduce the dimensionality of the data. The interaction network between the different Cd treatments and mineral elements was evaluated using a weight network diagram. Screening criteria for differentially expressed genes (DEGs) were | log2(fold change) | ≥ 2 and FDR < 0.05. Metabolites with variable influence on projection (VIP) values of > 1.0 and *P* < 0.05 were recognized differentially expressed metabolites (DEMs).

## Results and discussion

### Plant biomass of *S. Nigrum* under cd stress

**Fig. 1:**
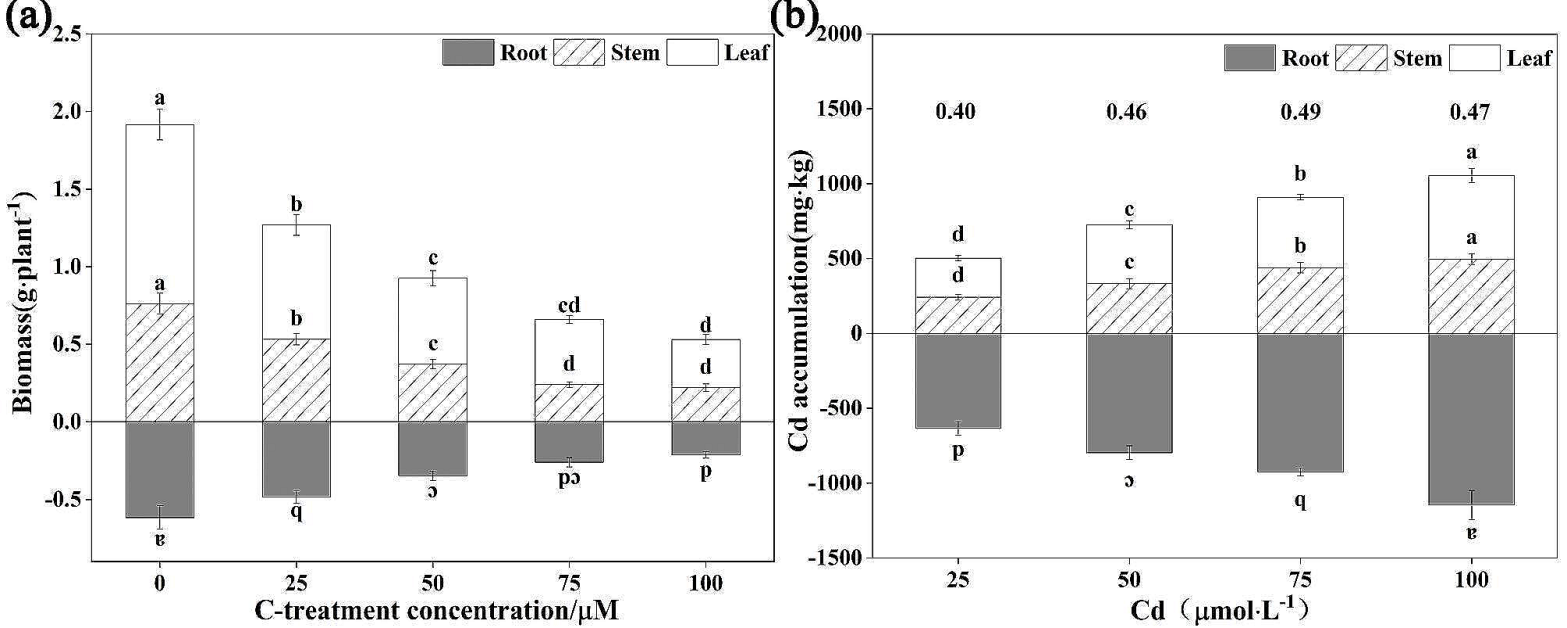
The influences of different Cd treatment on biomass growth **(a)** and Cd accumulation **(b)** in *S. nigrum*. The number at the top of the bar chart shows the TF value of Cd in *S. nigrum***(b)**. The values are the mean ± SD (*n* = 3). Different lowercase letters labeled on the columns show significant differences between different Cd treatments using Tukey’s test (*P* < 0.05)

Plant biomass is a crucial criterion that reflects tolerance to heavy metals (Sterckeman and Thomine, 2020). In this study, the biomass (dry weight) of roots, stems and leaves significantly decreased with increasing Cd concentrations (*P* < 0.05), which indicated that Cd inhibited normal plant growth (Fig. 1a and Fig. S1). Compared to the control treatments, the biomass in roots, stem and leaf sharply decreased by 65.41%, 71.09% and 73.11% when exposed to Cd under 100 µM stress, respectively. It was noted that the inhibition of the roots, stems and leaves biomass compared with other treatments was found at the 100 µM Cd treatment levels, which indicated that induced inhibition was gradually sharpened with increasing Cd concentrations. Our results are similar to previous studies demonstrating that Cd negatively affects plant growth, such as rice [44], wheat [27], and tomato [45], and its toxicity may be the comprehensive result of perturbation of the homeostasis of nutrient elements, water uptake and photosynthesis [26]. However, several studies proved that no reduction in *S. nigrum* biomass was found relative to their controls when Cd spikes were lower than 20 mg·kg^− 1^ in soil [46, 47]. Such different results may be due to the differences in the bioavailability or concentrations of Cd, and different cultivation conditions (hydroponics or soil) resulting in diverse threshold values of Cd tolerance in *S. nigrum*. Furthermore, these results also demonstrated that *S. nigrum* have high Cd accumulation and strong Cd tolerance at certain level, and it can be utilized as the main phytoremediation tool for Cd contaminated soil.

### The cd accumulation and translocation of *S. Nigrum*

The evaluation of heavy metal accumulation and translocation in plants is essential to disclose the mechanism of heavy metal tolerance in plants [48]. Therefore, the accumulation and translocation capacity of *S. nigrum* were assessed in the current study. The Cd concentration in *S. nigrum* different tissues showed a sharp upward trend with the gradient increment of solution Cd concentrations (Fig. 1b). The majority of Cd was observed to be fixed in the roots and the Cd concentration in three organs of *S. nigrum* declined in the order of root > leaf > stem under the same Cd treatment. In terms of Cd accumulation in the three tissues, under 25 ~ 100 µM Cd concentrations, Cd accumulation in roots ranged from 632.32 ~ 1 145.67 mg·kg^− 1^, in stems ranged from 241.63 ~ 495.08 mg·kg^− 1^, and in leaves reached 262.94 ~ 559.41 mg·kg^− 1^. In addition, the maximum Cd accumulation in different organs peaked level at 100 µM Cd treatments and excessive total accumulation compared to previous experiments performed in soil [35, 36, 49]. The results suggested that the roots immobilized more Cd than the stems and leaves parts, thereby reducing the toxic influence of Cd on the aboveground tissues in *S. nigrum*. These findings are similar to our previous studies demonstrated that the Cd concentration of different organs in *S. nigrum* was dose-dependently increased by external Cd addition [37].

Additionally, to better evaluate Cd distribution and translocation in *S. nigrum*, the TF values was calculated. As shown in Fig. 1b, the TF varied from 0.40 ~ 0.49, which was always less than 1.0, which indicates hyperaccumulation. The result did not agree with previous reports that the TF of Cd for *S. nigrum* was above 1.0 [50]. Previous researches have proved that *S. nigrum* has a great Cd uptake and translocation ability [35–37, 47, 51] It is possible that in the current study, *S. nigrum* was grown in hydroponic solution, which was different from previous experiments growing medium (in soil culture), and the concentration, mobility and bioavailability of Cd in the hydroponic system is much higher than that in soil conditions, leading to more Cd being easily immobilized in roots. In addition, *S. nigrum* maintained stable TF values, which slightly increased with the increasing Cd concentration, indicating that it can transport Cd uptake via roots to their aboveground parts for detoxification. A similar phenomenon was observed as described previously [52, 53].

### Other minerals contents, translocation factors and interaction

**Fig. 2:**
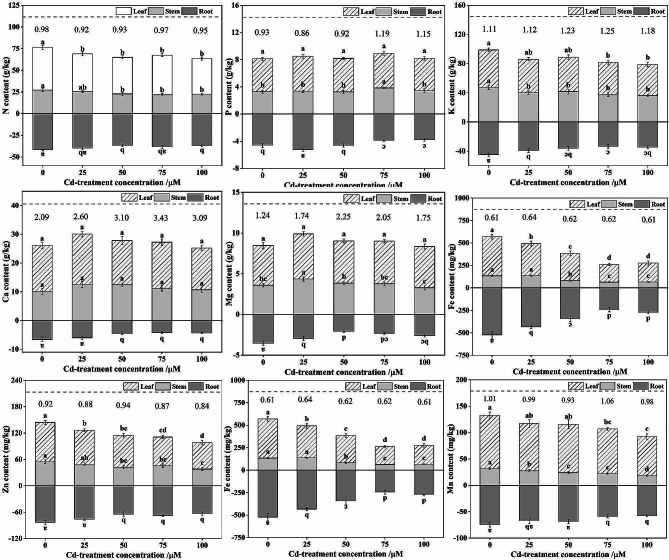
Effects of Cd on different mineral nutrients contents in *S. nigrum*. The number at the top of the bar chart shows the translocation factor of mineral nutrients in *S. nigrum*. Each value is the mean ± SD (*n* = 3). Different lowercase letters over bars indicate significant differences at *P* < 0.05

As reported, the metabolism process of heavy metals in plants can interact with mineral element metabolism, which can jointly mediate heavy metal uptake, distribution, and translocation [54]. The mineral element contents of *S. nigrum* are exhibited in Fig. 2, and the different Cd stress remarkably influenced the absorption of mineral elements. In terms of macro-elements, and the contents of N, K, Ca and Mg in roots gradually decreased with increasing Cd concentrations, respectively. For P content, the values slightly increased and then decreased, with growth rates of 0.88 ~ 14.45% and inhibition rates of 15.12 ~ 21.59%, respectively, compared to those of their control groups. Furthermore, Cd restrained the uptake of Fe, Cu, Zn and Mn via roots compared to the control, respectively. With regard to *S. nigrum* stem mineral nutrient absorption, Cd clearly interfered with N, K, Fe, Zn and Mn uptake as the Cd concentrations increased when compared to the control treatment, respectively. In contrast, at Cd concentrations of 25 ~ 75 µM, the Mg content in stem was increased by 4.65 ~ 21.15%, which decreased until the 100 µM Cd concentration. In addition, regarding mineral nutrients in leaves, there was a remarkable reduction in N, K, Fe, Cu, Zn and Mn with increased Cd in the nutrient solutions. Taken together, these results showed that Cd disturbed mineral element absorption and accumulation in *S. nigrum*, particularly at high Cd concentration.

Although the influence of Cd on macro- and micro- nutrient absorption, translocation, distribution and accumulation in plants have been investigated, whether the Cd inhibits mineral element uptake or increases mineral element accumulation varies depending upon Cd concentrations in environment [22]. Cd^2+^ can compete with plant essential nutrients for the binding sites of membrane transporters on the roots surface, and then it enters roots cells by occupying Ca, Mg, Fe, Zn and other metal cation channels to affect the uptake of mineral elements [5, 11]. Therefore, this result suggested that the electrochemical potential gradient of the *S. nigrum* root plasma membrane changed under Cd stress, especially at high Cd levels, which can affect the mineral elements absorption and accumulation. Similar results were observed in ryegrass [24], rice [22] and tomato [55].

**Fig. 3:**
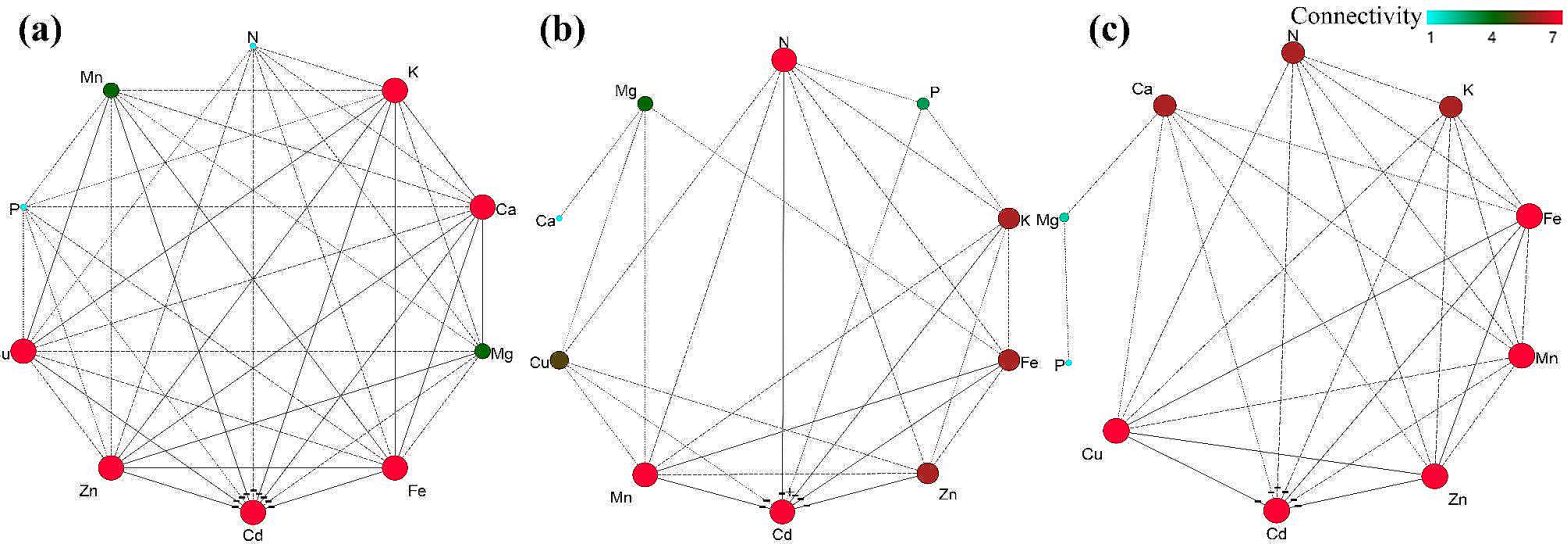
The interaction network analysis of mineral elements in *S. nigrum* roots **(a)**, stems **(b)** and leaves **(c)** under Cd concentrations treatments. The redder the node color and the larger the circular node in the weight network analysis, the greater the element’s connectivity in the weight network, that is, the more distinct the influence of this element on other nutrient elements. The different lines between the mineral elements (dotted and solid) represent the degree of correlation. “+” represents there is a positive correlation between the two elements, and “-” represents there is a negative correlation between the two elements

To evaluate the translocation capacity of these mineral elements, the TFs of the mineral elements were calculated. As shown in Fig. 3, Cd stress had influences on the TF values of these mineral elements. Compared with CK, the TF value of N, Cu, Zn and Mn decreased slightly with increasing Cd supplementation, indicating that Cd inhibited the translocation of these nutrient elements to the aboveground parts. In contrast, the TF values of P, K, Ca, Mg and Fe exhibited a trend toward an increase firstly, and then followed by a decrease with increasing Cd addition. These results indicated that Cd remarkably perturbs the nutrient elements metabolism of *S. nigrum* under Cd stress, and the extent of perturbation was associated with Cd doses. Interestingly, we observed that the contents of N, K, Fe, Cu, Zn, and Mn were lower than those in the CK aboveground parts, while the TF values of these nutrient elements were not much different from those in the CK group. This means that although Cd stress inhibited the uptake and accumulation of nutrients in *S. nigrum* roots, to maintain its normal life activities, the nutrients in the roots were transported to the aboveground parts as much as possible. The strategy may be the Cd tolerance mechanism adopted by *S. nigrum*, which agreed with previous findings that the TF value of some mineral elements for rice was not significantly changed with increased Cd concentrations [22].

Furthermore, we also found that although the contents of P, Ca and Mg in roots was reduced, the contents of these nutrients in the aboveground parts were increased, and the TF values of these three elements were higher than those in the CK group, especially for Ca and Mg. For P elements, P can mitigate the toxic influence of Cd on plant by (1) increasing the pectin and hemicellulose content of the plant cell walls, which enhances the Cd immobilization ability, (2) forming chelates/precipitants with stable structures (for example, Cd_3_(PO_4_)_2_), and (3) co-sorbing P and Cd as an ion pair [18, 25]. Hence, it has to be noted that the increased content of P in the aboveground parts may be an underlying detoxification mechanism of Cd in *S. nigrum*. Meanwhile, possible mechanisms for alleviating Cd toxicity by Ca are as follows: (1) Cd ions have an ionic radius similar to that of Ca ions and probably competes for the same Ca transporters, channels or binding sites, (2) Ca preserves membrane stability and cell integrity, which could hamper Cd from entering the roots cell, and (3) Ca primarily exists in the chemical form of Ca-pectinate in plant cell wall, which helps to augment the thickness and stability of cell wall, enhance the ability of cell wall to retain, sequester and accumulate Cd, and thus reduce the Cd content in intracellular [25, 56]. It should be noted that these mechanisms can occur simultaneously. In this study, the increase in the TF-Ca value of *S. nigrum* probably plays a positive role against Cd toxicity, which has crucial function some metabolic processes. Indeed, similar effects were found in pakchoi and *Sedum alfredii* [57, 58]. Mg is one of the essential elements for chlorophyll synthesis and also deemed to an activator for more than 300 enzymes [25, 59] Some studies have reported the interaction of Mg and Cd, for example, Mg deficiency can increase Cd immobilization in different tissues of *Salix viminalis*, and the uptake and accumulation of Mg in brown rice was reduced under high Cd level [60]. In this study, we speculate that this may be a detoxification strategy of *S. nigrum* by facilitating the translocation of Mg from roots to aboveground parts, and thereby effectively reducing the Cd-derived photosynthesis inhibition. All of the results above indicated that the intense tolerance abilities of *S. nigrum* under Cd conditions could be related to the regulation of mineral element absorption.

To further evaluate the interaction network between Cd and nutrient elements in different *S. nigrum* tissues, the weight network analysis was implemented (Fig. 3). The result showed that Cd exhibited a significant negative correlation with mineral elements, and a competitive uptake relationship was obvious between Cd and nutrient elements, especially with K, Ca, Cu, Zn and Fe, suggesting that the nutrient elements absorption pathway of *S. nigrum* could be related to the absorption and translocation of Cd. In addition, Cd, Fe, Zn, Cu and K showed higher connectivity with other nutrient elements, suggesting that they were at a crucial node of the nutrient element metabolism regulation network. This may also be the reason that the TF value of these elements in *S. nigrum* did not significantly change under the five Cd stresses. One point worth mentioning is that, Cd exposure results in disturbances in the normal mineral metabolic profiles of different tissues of *S. nigrum*, revealing that aberrant mineral elements metabolism could be one of the primary reasons for uncommon different tissues of *S. nigrum* growth, while the difference in the different organs response to Cd was tightly connected with the metabolism of mineral nutrients. This finding is notable because Lai et al. [19] found a distinct interaction between Cd and nutrient elements in *Ipomoea batatas* roots, and Khaliq et al. [22] reported that obvious antagonism was found between Cd and Fe, Zn, and Ca. Based on the aforementioned results, it is feasible to enhance the hyperaccumulators remediation efficiency on Cd-contaminated soil through regulating the application of mineral nutrients to some extent, while the molecular mechanism of the mineral and Cd interaction needs further elucidation.

### Response of biochemical indexes of *S. Nigrum* roots and leaves under cd stress

#### Photosynthetic pigments

**Fig. 4:**
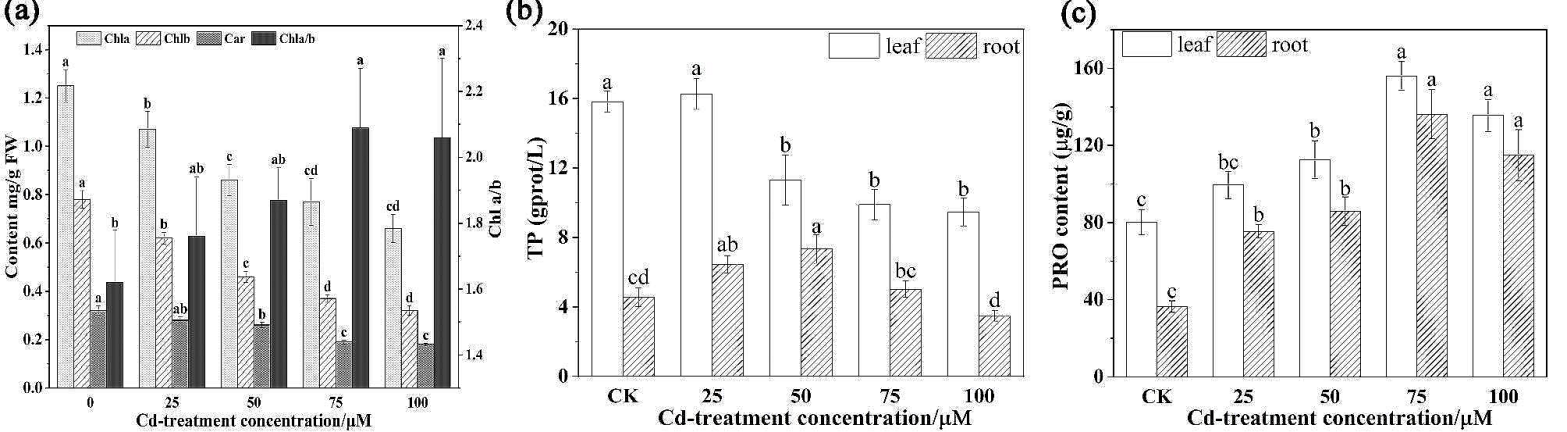
The photosynthetic pigment **(a)**, total protein **(b)** and proline **(c)** contents in *S. nigrum* under 0, 25, 50, 75 and 100 µM Cd concentrations in hydroponic solutions for 7 d. Bars indicate the mean of 3 independent replicates ± SD. Different letters over bars indicate significant differences at *P* < 0.05, according to Tukey’s test

The growth of plants in excess heavy metals conditions such as Cd influence the photosynthetic pigments synthesis process, and changes in photosynthetic behavior can also be used to assess plant in responses to heavy metal stress [6, 61]. The photosynthetic pigment content dramatically reduced with increasing Cd concentration, specially at 100 µM Cd treatment, the content of Chl a, Chl b and Car showed the highest reduction of 47.2%, 58.97% and 44.75%, respectively, compared with the control (Fig. 4a). According to previous studies, Cd can influence the photosynthesis pathway by damaging the ultrastructure of chloroplasts, inhibiting the activity of photosynthetic pigments, and hampering the synthesis of photosynthetic pigments. Meanwhile, several studies also observed that a low dose level of Cd could enhance plant photosynthesis capacity [62, 63]. This probably depends on different plant species, Cd concentrations, and the timing of exposure. In addition, interestingly, the trend of the Chl a/b ratio increased with increased Cd concentrations; among them, the value of Chl a/b was obviously increased by 29.01% under 75 µM Cd concentrations compared to control treatment, and the major reason was that the decrease in core pigments Chl a was lower than Chl b. The Chl a/b ratio, a reflection of plant light-use efficiency, representing the proportion of stacked versus unstacked membrane domains [63]. Therefore, the results revealed that Cd treatment appropriately enhanced the value of Chl a/b in *S. nigrum*, which resulted in alleviating thylakoid damage and photoinhibition, although high concentrations of Cd will disrupt photosynthesis. This was similar with previous reports [64, 65].

#### Total protein and proline

Protein is one of the most important basic substances for plants to maintain metabolism and function and can be used as an important indicator to detect their physiological status [65]. Figure 4b illustrated that the content of total protein (TP) in both leaves and roots firstly raised and then declined with the gradual increase in Cd concentration. The root and leaves TP content of *S. nigrum* under 100 µM Cd treatments was always significantly lower than the CK treatment, respectively. These findings revealed that low Cd concentrations (< 25 µM) could stimulate an increase in TP content, both in roots and leaves, while the adverse effect on the TP content and the degree of influence increased with increasing Cd concentration. The increase in protein content in *S. nigrum* was most likely induced by stimulation of the synthesis of stress proteins, such as heat shock proteins (HSPs). However, a decrease in protein content was observed under high Cd concentrations, which can promote ROS formation, leading to the degradation of protein. This phenomenon also complies with our previous findings [35].

Proline is a nonessential amino acid biosynthesized in chloroplasts and plant-cell cytoplasm that can protect macromolecules, sustain the cell membrane stability, and decrease ROS accumulation under abiotic stress [6, 14, 15]. The content of proline (PRO) in the leaves and roots is shown in Fig. 4c. The variation trend first augmented and then declined slightly with increased levels of Cd. The PRO content in roots and leaves under 75 µM Cd treatment was 3.73 and 1.95 times higher than the control, respectively. In addition, more PRO was accumulated in leaves than in roots under the same Cd treatment, suggesting that leaves are more sensitive than roots in response to PRO. Some previous studies have also demonstrated that proline accumulation is an adaptive response of plants under Cd exposure, for example in alfalfa, wheat and *Lolium perenne* L [24, 27, 66]. In summary, the PRO overproduction in *S. nigrum* was a constituent of the Cd detoxification defense strategy.

#### Oxidative responses and antioxidant systems

**Fig. 5:**
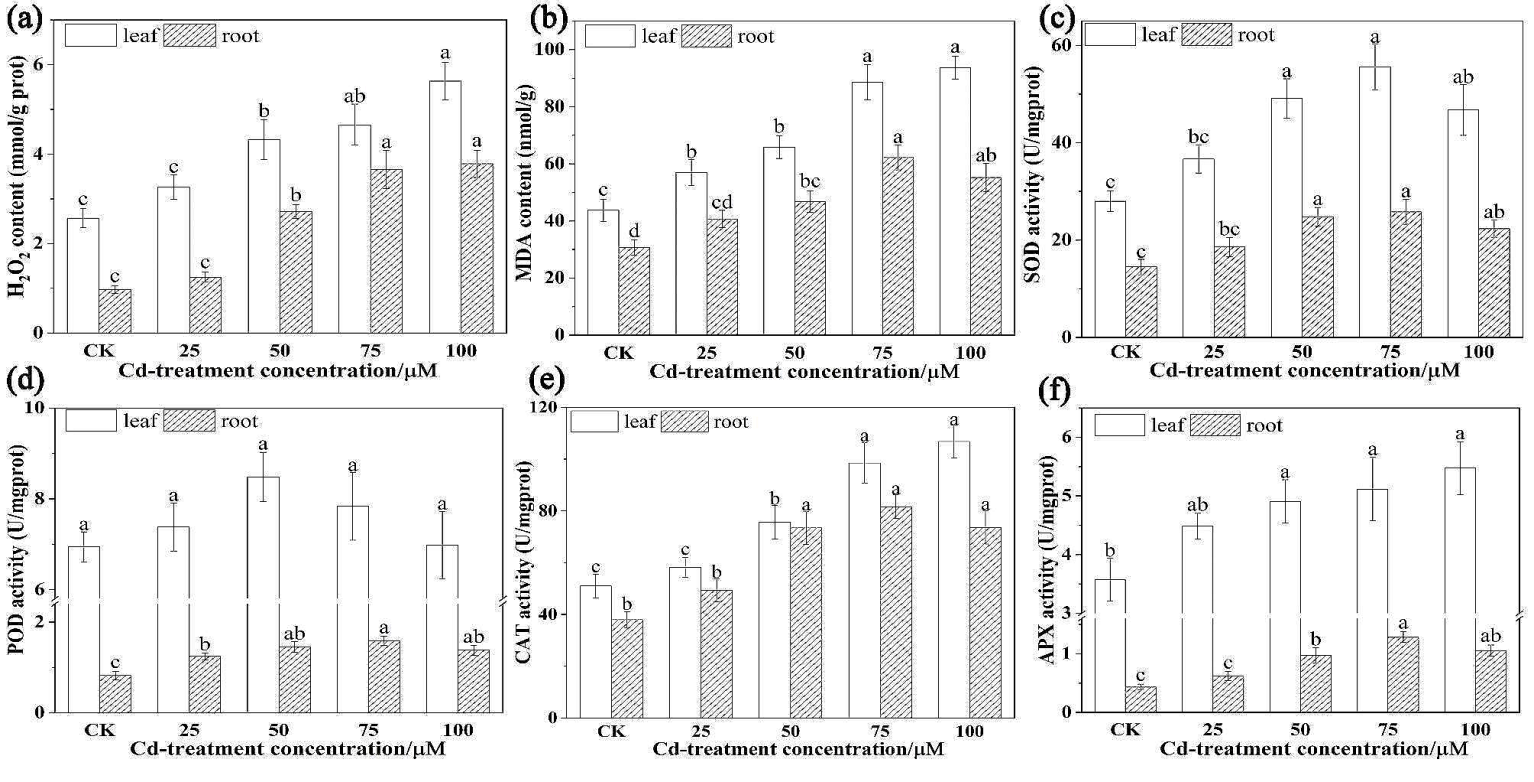
The H_2_O_2_**(a)**, MDA **(b)**, SOD **(c)**, POD **(d)**, CAT **(e)** and APX **(f)** contents in *S. nigrum* in response to five Cd concentrations (0, 25, 50, 75 and 100 µM) in hydroponic solutions for 7 d. Bars indicate the mean of 3 independent replicates ± SD. Different letters over bars indicate significant differences at *P* < 0.05, according to Tukey’s test

High concentrations of Cd inhibit the activity of various metabolic cycles and non-redox active metals that cause the ROS overproduction, for instance H_2_O_2_, superoxide radicals (O_2_‧^−^), and hydroxyl radicals (OH‧^−^) in the cytosol [6]. Moreover, it damages the integrity and selective transport system of plasma membranes, causing lipid peroxidation and inducing lipid peroxide breakdown to produce, for instance, MDA, which results in oxidative cellular damage [15]. In this experiment, compared to the control, the H_2_O_2_ and MDA contents were significantly elevated in Cd-treatment roots and leaves, besides no significant boost in H_2_O_2_ at 25 µM Cd exposed roots and leaves, and in MDA at 25 µM Cd exposed roots (Fig. 5a and b). The results regarding in the roots and leaves under 100 µM Cd treatment was 3.96 and 2.19 times higher than the CK, respectively (Fig. 5a). Similarly, MDA contents in 75 µM Cd treatment roots and 100 µM Cd treatment leaves showed 2.11 and 2.03 times higher than the CK, respectively (Fig. 5b). Our results indicated that Cd toxicity was magnified, and the degree of lipid peroxidation and cell membrane damage was aggravated with increasing Cd concentration. Additionally, the present study findings were also consistent with previous studies, suggesting that when plants are abruptly exposed to Cd stress, the contents of H_2_O_2_ and MDA are immediately elevated [8].

In general, ROS production in plants is the normal reaction under heavy metal stress. However, ROS overproduction in plant tissues is toxic, and plants try to avoid its adverse effects by adopting various defense mechanisms, which include efficient antioxidant defense system activation (e.g., SOD, POD, CAT and APX) to overcome this overproduction [5]. For instance, SOD starts the disproportionation of superoxide radicals to form oxygen and less toxic hydrogen peroxide, which are subsequently converted into water through the regulation of APX and POD. Furthermore, CAT can also catalyze the decomposition of hydrogen peroxide into water and oxygen [5]. Consequently, the several antioxidant enzymes activities were further measured to assess the response of *S. nigrum* under Cd stress.

As shown in Fig. 5c-f, the activity of antioxidant enzymes was significantly improved and showed similar response patterns with increasing Cd levels, both in leaves and roots, relative to the control. The SOD activities reached maximum under 75 µM Cd treatment, which was remarkably increased by 98.57% and 78.31% compared to the control, and then declined afterward, both in leaves and roots, respectively (Fig. 5c). The activity of POD at the 50 µM and 75 µM Cd treatments was the highest in leaves and roots, and was approximately 22.14% and 93.09% higher than that in CK, respectively (Fig. 5d). Similarly, we observed that the activity of CAT under 100 µM Cd treatment was the highest at approximately 106.80 U/mg protein (prot), followed by that in 75 µM at approximately 98.49 U/mg prot in leaves, and in the 75 µM Cd treatment, it was the highest at approximately 81.63 U/mg prot, followed by that in 100 µM at approximately 73.56 U/mg prot in roots, respectively, which was remarkably different from CK (Fig. 5e). Furthermore, a 53.40% increase in leaf APX activity under 100 µM Cd treatment and a 196.62% increase in root APX activity under 75 µM Cd treatment were observed compared to CK (Fig. 5d). Interestingly, we also found that the antioxidant enzyme activities decreased slightly under 100 µM Cd concentration, both in leaves and roots relative to 75 µM Cd concentration, indicating that the enzymes have an activity threshold. Simultaneously, the antioxidant enzyme activity of leaves was always greater than that of roots regardless of whether the plants were treated with Cd or CK, indicating that the degree of antioxidant enzymes in response to Cd stress varies among different *S. nigrum* tissues.

Notably, these data indicate that *S. nigrum* can effectively ensure the normal eco-physiological activities of plant tissues by strengthening the synergistic effect of various antioxidant enzymes, which establishes a defense system against ROS by Cd stress. Nonetheless, the excessive production of ROS in *S. nigrum* cannot eliminated in time, and the antioxidant defense system will collapse when the environmental Cd concentration is too high, thus leading to a decline in antioxidant enzyme activity. In conclusion, these results were also in line with previous studies where the antioxidant enzyme activities in mulberry, *Dendrobium officinale* and ryegrass were improved with increasing Cd concentration [11, 24, 67].

### Multivariate analysis

**Fig. 6:**
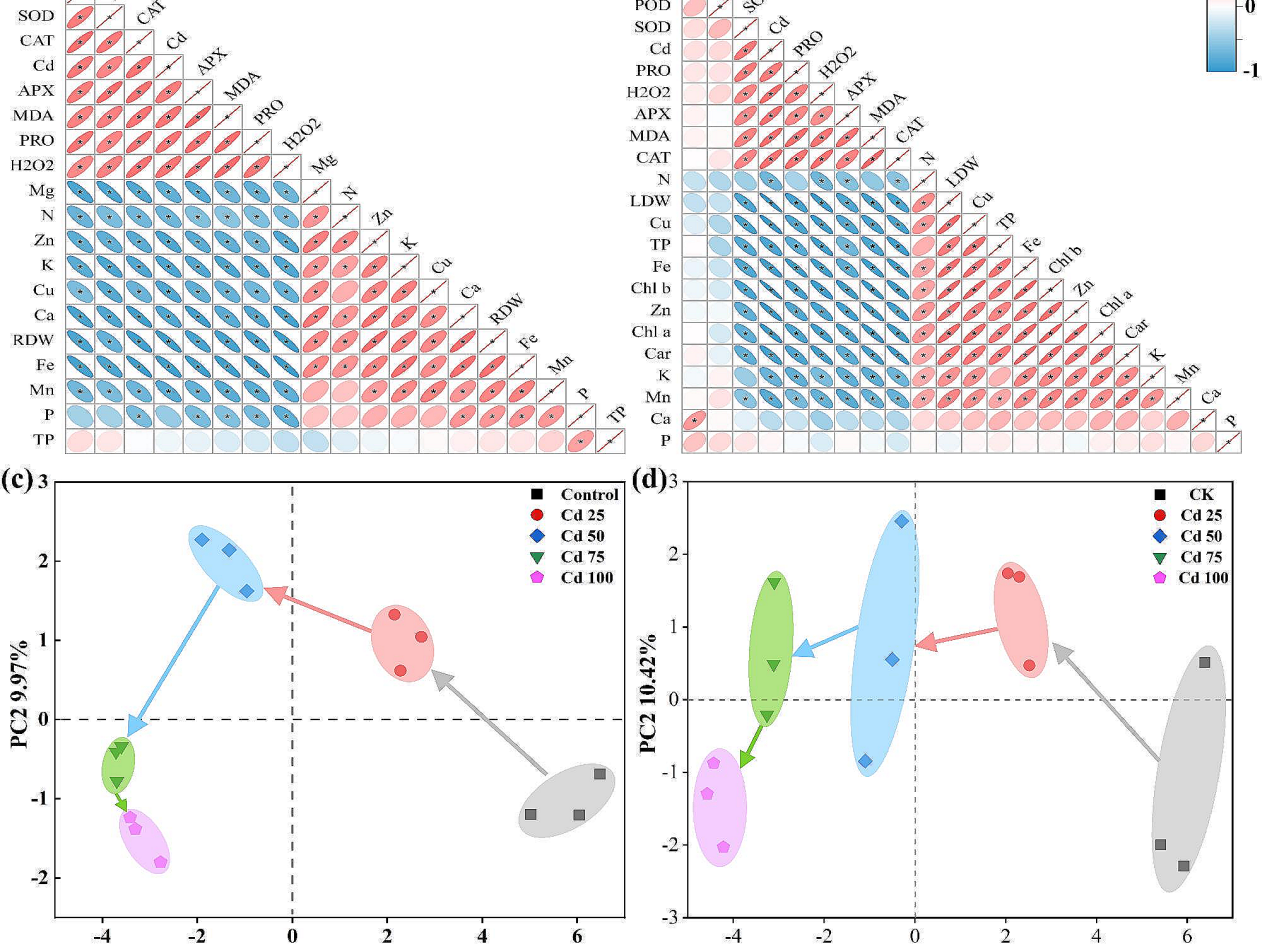
The correlation between morphophysiological properties, mineral elements and Cd concentrations of roots **(a)** and leaves **(b)** in *S. nigrum*. * indicates significant correlations at *P* < 0.05. The PCA results of the morphophysiological properties and mineral elements in roots **(c)** and leaves **(d)**

To visualize the comprehensive interaction relationship between Cd and the investigated parameters, the Pearson correlation coefficient was assessed. Correlation analysis of the investigated parameters in roots and the Cd content was significantly and positively related to the antioxidant enzyme activities, PRO, H_2_O_2_ and MDA, but negatively related to the RDW and mineral elements, except P and TP (Fig. 6a). Similarly, the Cd content in leaf was remarkably and positively related to PRO, H_2_O_2_, MDA, and the activity of SOD, APX and CAT, but negatively related to TP, LDW, photosynthetic pigments and mineral elements, except P and Ca (Fig. 6b). Interestingly, we further observed that the mineral elements were positively correlated with each other, and the indicators of physiology and biochemistry were also positively correlated with each other, both in roots and leaves, while they were mostly negatively correlated with each other. The results are mainly in line with the aforementioned discussion and demonstrate that Cd has a strong influence on plant physiological properties.

To further investigate the responsive patterns of all the research parameters determined here to Cd at different concentrations, PCA was performed to reduce the information and dimensionality of the initial data. The PCA results were shown in Fig. 6c and d, unambiguous separation in principal component 1 and principal component 2 (PC1 and PC 2) of physiological metabolism on account of under different Cd treatments, the first two principal components explained 84.34% and 78.83% of the total variation in the root and leaf entire data set, respectively. The results showed that the cluster position of the investigative parameters in the PCA map was distinctly moved with raising Cd from 0 to 100 µM, which demonstrated that Cd stress significantly interfered with the normal growth and development, and physiological metabolism of *S. nigrum*, and the degree of interference was associated with the Cd level. However, more studies are required to investigate the interaction of Cd and other variables at the molecular level, which may be more interesting.

### Effects of Cd exposure on the root transcriptomics and metabolomics

Our morphophysiological investigation indicated that Cd disturbed the mineral nutrients metabolism, antioxidant systems, photosynthesis and plant growth. To further understand and elucidate the influence of different Cd concentrations and the molecular mechanism associated with Cd detoxification on *S. nigrum*, previous transcriptome and metabolome data were selected for analysis relative to those processes [32].

#### DEGs related to mineral nutrient and cd transport or detoxification

As mentioned above, mineral nutrient metabolism in plant roots can interact with Cd absorption, distribution, accumulation and translocation, and physiological stress response [18, 22]. Plant roots can uptake mineral elements and heavy metal ions by ion channels and transporters for necessary metal ions, including Fe^2+^, Ca^2+^, and Zn^2+^ [18, 57]. The regulatory networks of this process may be rather complicated; however, the related functional genes might explain the Cd-induced expression patterns of mineral nutrient metabolism. Therefore, in view of this, transcriptome sequencing of the *S. nigrum* roots was analyzed and the differentially expressed genes (DEGs) between the control and Cd-treated group were identified based on |log2FoldChange|> 1 and p-value< 0.05 by using the DESeq R package. A total of 37 DEGs (Fig. S2a and Table S2) involved in mineral element ion transporters from the common DEGs (666 upregulated and 2586 downregulated, Table S3) were identified. Among these, Cd accumulation remarkably changed the normal expression patterns of some genes related to the inorganic ion transporters and metabolism of N, P, K, Ca, Fe, Cu and Zn, which might have resulted in a metabolic disbalance of these mineral elements (Table S2).

For instance, N is a crucial nutrient element for plant growth, and its uptake, transport and accumulation in plants are correlated with nitrate nitrogen, ammonium nitrogen and polypeptide transporters [68, 69]. In the current study, a total of 7 (1 upregulated and 6 downregulated) DEGs were related to nitrate and ammonium transporters (Fig. S2a and Table S2), indicating that Cd stress disturbed the uptake, translocation and accumulation of N in *S. nigrum* roots. For others macro-elements, such as P, K, and Ca, there are 4 DEGs (2 upregulated and 2 downregulated), 11 DEGs (1 upregulated and 10 downregulated) and 6 DEGs (2 upregulated and 4 downregulated) were identified, respectively (Fig. S2a and Table S2). Likewise, for microelement Fe, Cu and Zn, some DEGs were also identified (Fig. S2a and Table S2). The result was consistent with a previous study, which also found that the genes related to the transport of mineral elements were differentially expressed in mulberry under Cd treatment [11]. In summary, Cd treatment intervened in the mineral element metabolism balance of *S. nigrum* roots, especially at high concentrations, and the aforementioned changes in the expression pattern of functional genes could also explain Cd-induced plant toxicity.

Generally, Cd ion enters root cells via ion channels and transporters for essential elements, like Ca, Fe and Zn [57]. Therefore, we screened 44 DEGs involved in Cd absorption and transport from the common DEGs, as shown in Table S4. Previous studies have reported numerous transporter genes related to Cd translocation, such as the *ZRT/IRT*, *NRAMP*, *COPT*, *YSL* and *ABC* families [11, 70]. *ZRT/IRT* are proteins belong to the *ZIP* transporter family and they are primarily related to cation translocation across the cell membrane, implicated in Cd root-to-shoot transport, contributed to Cd detoxification [11]. In the present study, 2 upregulated DEGs were found, illustrating that they are critical for the absorption and translocation of Cd in *S. nigrum* roots. Natural resistance-associated macrophage protein (*NRAMP*) contributes to the uptake, translocation, and detoxification of heavy metals in plants [23]. Here, we found 1 upregulated DEGs belonging to the *NRAMP* family. Previous studies have reported that *NRAMP 1, NRAMP* 3, *NRAMP* 4 and *NRAMP5* in rice, tomato and *Brassica napus* participate in the Cd detoxification process [4, 45]. The upregulated expression of these DEGs may suggest that they can effectively accelerate Cd transport and improve the Cd detoxification in roots. Similarly, the oligopeptide transporter (*OPT*) family possesses approximately 16 transmembrane domains that can transport numerous substrates in plants [71]. In this study, 2 upregulated DEGs involved in this family were found. Furthermore, interestingly, we also found 1 upregulated DEGs belonging to the yellow stripe-like transporter family, which has an *OPT* domain transporting its substrates from either the extracellular environment or an organelle into the cytosol [72]. They are related to the transport and homeostasis of Fe, Zn, and Cd, etc [72]. Overall, these results implied that they might play a crucial role in Cd detoxification and resistance of *S. nigrum* responses to Cd stress.

In addition, *ABC* family transporters belong to a ubiquitous superfamily that regulates all sorts of molecular transport approaches [72]. In the current study, 38 DEGs related to *ABC* transporters were identified and the number was the highest (Table S5). They were involved in *ABCA*, *ABCB*, *ABCC* and *ABCG* subfamily members and showed significant changes. Previous some researches have confirmed that *ABC* transporters play a crucial role in heavy metals detoxification in plants, for example, *ABCB* transporters can enhance the resistance of *Triticum polonicum* L., *ABCC* transporters can transport PC-Cd or GSH-Cd from the cytoplasm to the vacuole, leading to detoxification, and *ABCG* transporters can transport intracellular Cd across the membrane to the extracellular space, thus enhancing plant tolerance to Cd [73]. These findings further elucidated that *S. nigrum* roots adopt the strategies of pumping out excessive intracellular Cd to the extracellular space or transporting it to inactive areas (such as vacuoles) to improve their tolerance to Cd.

#### DEGs related to the antioxidant system

Multiple studies have showed that Cd toxicity induces ROS overproduction, including H_2_O_2_ and leads to oxidative stress in plants, which is a widespread phenomenon under Cd stress [5]. Cellular antioxidative enzymes are commonly stimulated to help plants resist oxidative damage by eliminating ROS [6]. Simultaneously, their gene expression levels and patterning were also changed. According to the antioxidant enzyme activity of this study, the corresponding genes should be explored to evaluate the effects on *S. nigrum* under Cd stress. There were evident alterations in 20 DEGs related to antioxidant enzyme genes, including SOD, CAT and POD, while the DEGs involved in APX were not found (Fig. S2b and Table S5). Among these 20 DEGs, 6 were upregulated and 14 were downregulated. The results suggested that the expression levels of these DEGs changed, and leading to alterations in the activities of antioxidant enzymes, thereby influencing the Cd-induced ROS production. However, with the aggravation of Cd stress, the intracellular antioxidant enzyme system has no time to scavenge ROS, and when it accumulates to a certain extent, it results in damage to the antioxidant enzyme system. Thus, this is a process of constantly maintaining and destroying the balance of plants between elimination and ROS generation. It can be speculated that the tolerance of *S. nigrum* was associated with Cd-induced expression of DEGs related to antioxidant enzymes under Cd stress. This result also revealed the molecular mechanism of antioxidant enzymes activities in *S. nigrum* under Cd condition.

#### Validation of qRT-PCR

**Fig. 7:**
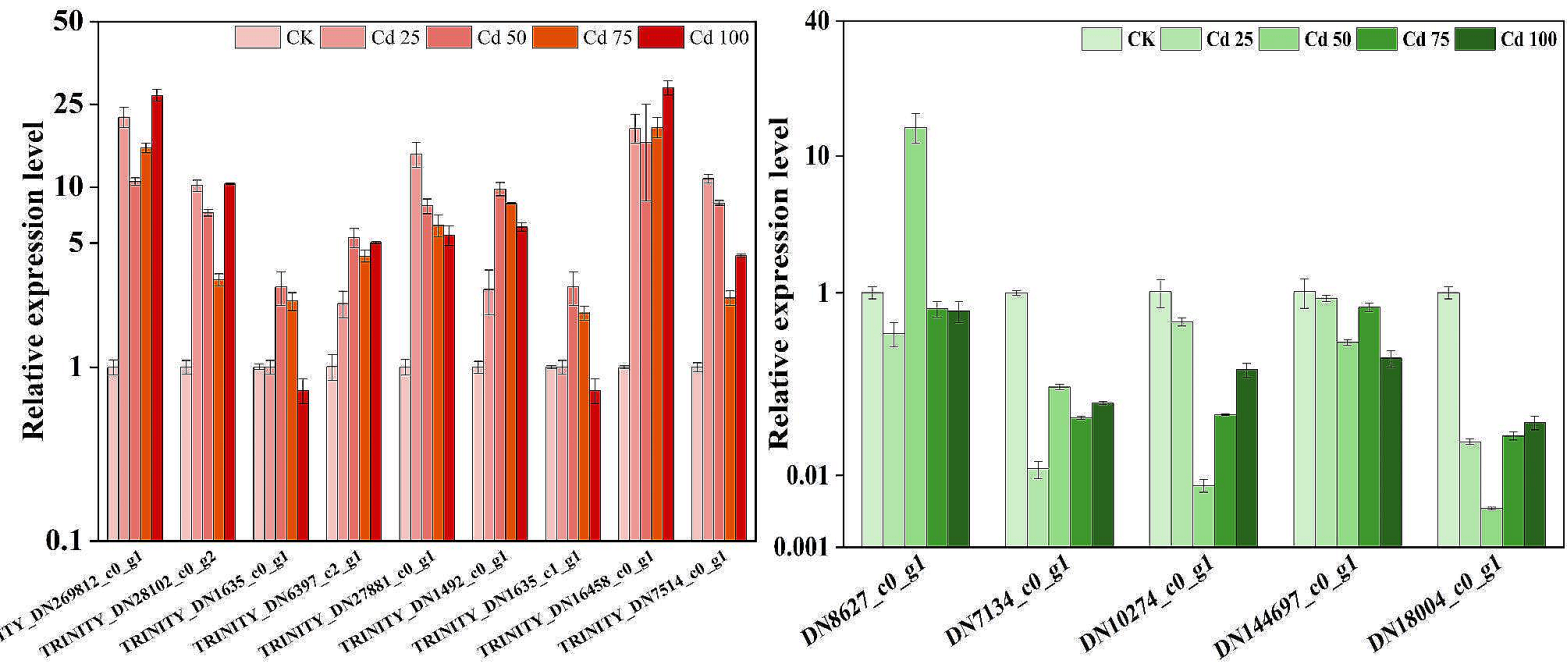
Reliability of DEGs expression by qRT-PCR analysis of fourteen DEGs. The qRT-PCR values are means ± SD (*n* = 3). Red represents upregulated genes and green represents downregulated genes

To clarify the dependability of the RNA-seq results, the fourteen DEGs related to mineral nutrient metabolism, Cd transport and antioxidative stress was chosen for qRT-PCR. The expression levels of fourteen genes were in agreement with the sequencing data, suggesting the reliability of the RNA-seq data (Fig. 7).

#### Effects of Cd-exposure on root metabolome profiling

**Fig. 8:**
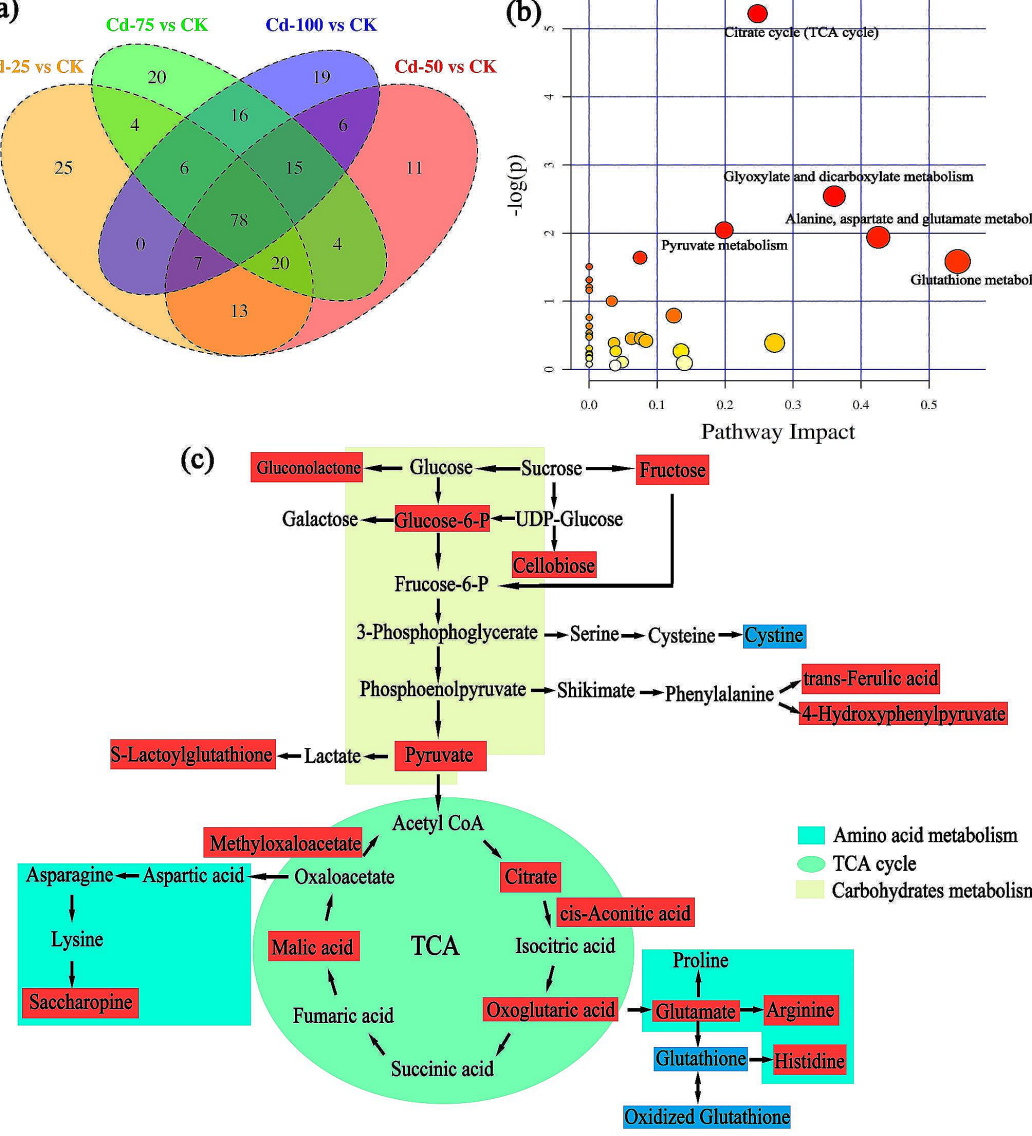
**(a)** Venn diagram of commonly and exclusively expressed DEGs in the comparison of the control and treatment groups. **(b)** Bubble plot of significantly common metabolic pathways by metabolomics of *S. nigrum* roots exposed to five Cd concentrations. **(c)** Summary of the significantly changed metabolic pathways in *S. nigrum* roots under five different Cd concentration exposures. *Note***(b)** Each circle denotes a metabolic pathway, with red color indicating higher - log(p) value and yellow color indicating lower - log(p) value. The size of the circle indicates the pathway impact value in the topological analysis. **(c)** The red background is upregulated DEMs, and the blue background is downregulated DEMs

In metabolomic, differential metabolites can reflect the metabolic processes and physiological mechanisms of plants [29]. Based on our previous metabolome profiling, we first screened the differentially expression metabolites in the four comparisons (e.g. CK vs. Cd 25, CK vs. Cd 50, CK vs. Cd 75, CK vs. Cd 100) using a *t*-test. According to the Venn diagram, a total of 153, 154, 163 and 147 DEMs were identified and semi-quantified in the four comparisons, including 118, 103, 113 and 88 metabolites with upregulated expression and 35, 53, 50 and 59 metabolites with downregulated expression, respectively (Fig. 8a and Table S6). Interestingly, differential metabolomics responses between the different Cd and CK treatments were remarkable in amino acids, carbohydrates, lipids, nucleotides and some plant growth substances, both quantitatively and compositionally. Notably, there were 78 DEMs under each comparison group mainly consisting of 13 amino acids (16.67%), 12 carbohydrates (15.38%), 10 lipids (12.82%), 10 nucleotides (12.82%), 7 xenobiotics (8.98%), 6 cofactors and vitamins (7.69%), 1 carboxylic acids and derivatives (1.28%), and 19 others (24.36%), indicating that those DEMs play essential roles in Cd regulation and detoxification in *S. nigrum* roots (Table S7). Hierarchical clustering analysis was utilized to explore the DEMs (78) expression patterns in *S. nigrum* roots under Cd treatments, and the results are exhibited as heatmaps (Fig. S3). Diverse colors in the heatmap represented diverse variety of metabolic modes, and the biological function of the identified DEMs could be speculate by the heatmaps. The results showed that after different Cd treatment groups were converged, they clustered with CK, indicating that Cd stress varied the *S. nigrum* root metabolism pathways; meanwhile, there were few diversities in the metabolic changes in the four Cd treatment groups, which suggested that some metabolic response processes of roots was similar under these treatments. Taken together, these results are anticipated due to plants under Cd conditions sustain their cellular homeostasis through generating and regulating numerous pivotal metabolites, for instance carbohydrates, amino acids and lipids.

#### Metabolic pathway analysis of DEMs in *S. Nigrum* under cd treatments

Furthermore, based on previously published data, a comprehensive KEGG pathway analysis was performed on DEMs in *S. nigrum* roots; consequently, 6, 6, 9 and 4 pathways were annotated for *S. nigrum* under different Cd stresses with criterion of *P* < 0.05 and impact score > 0.1, respectively (Table S8). In addition, the 78 detected common DEMs were subjected to MetaboAnalyst for metabolic pathway analysis. Interestingly, five significant metabolic pathways were identified and screened, including “citrate cycle (TCA)”, “alanine, aspartate and glutamate metabolism”, “glyoxylate and dicarboxylate metabolism”, “pyruvate metabolism” and “glutathione metabolism”, which are mainly involved in amino acid and carbohydrate metabolites (Fig. 8b). It can be speculated that with the increase in Cd concentration, the *S. nigrum* root may gradually activate different metabolic pathways to cope with Cd stress; simultaneously, indicated that these metabolic pathways were very momentous to response to Cd and alleviate the Cd toxicity.

Some studies have demonstrated that heavy metals directly cause an imbalance in photosynthetic metabolism, carbohydrate metabolism, amino acid metabolism, and nucleic acid metabolism, leading to the inhibition of growth and development of plants [74, 75]. Amino acids are generally necessary for the biosynthesis of proteins and are related to the regulation of osmotic pressure, and they play crucial roles in plant oppositions to heavy metal stresses [75]. In the present study, Cd stress restrained the biosynthesis of amino acids like L-cystine, glutathione, and oxidized glutathione in *S. nigrum* roots, and facilitated the synthesis of L-histidine, L-glutamic acid, L-arginine, and saccharopine, which could have been associated with the function of amino acids. For example, L-cystine is the precursor of glutathione (GSH), while GSH offers precursor for phytochelatines (PCs) biosynthesis, and is also involved in the scavenging ROS and Cd chelation [76]. Hala Rajab et al. [77] found that the increase in the levels of cysteine and glutathione can enhance *Brassica napus* L. tolerance to Cd stress. This was inconsistent with our results, which might be due to the different of plant species, exposure times and concentrations. In addition, other amino acids also were important for sustaining the growth and heavy metal detoxification of plants. For example, histidine was confirmed to play a crucial role in adjusting the biosynthesis of other amino acids, and in the detoxification, chelation and transport of metal ions [76]. These results also proved that L-histidine was significantly upregulated with increasing Cd concentrations; thus, the synthesis of L-histidine seems to be closely related to respond to Cd conditions. Likewise, recent studies verified that arginine metabolism can generate concerned osmotic regulating substances, which can withstand osmotic imbalance under Cd stress [29]. In the current study, L-arginine was remarkably upregulated, indicating that *S. nigrum* roots might have adjusted their penetration balance via arginine metabolism, and the osmotic regulation substances production via other metabolic pathways, thus enhancing their adaptation and tolerance to Cd. However, it is essential to further elucidate the metabolites of amino acid, illustrate their function in the Cd resistance and accumulation, and stimulate these metabolites biosynthesis using molecular tools to facilitate the phytoremediation efficiency of *S. nigrum*.

Additionally, carbohydrates are the primary energy storage substances and can be adjusted under heavy metal stress, as their content was once deemed to be an indicator of a plant’s physiological state [31]. In this study and combined with our previous data, the expression level of carbohydrates was observed, such as fructose, pyruvic acid, citric acid, L-malic acid and cis-aconitic acid, which are mainly related to glycolysis, the TCA cycle, and other metabolic pathways (Table S7). Among them, the expression of fructose, glucose-6-phosphate and pyruvic acid related to glycolysis metabolism was significantly upregulated with increasing Cd concentrations, indicating that *S. nigrum* can provide more energy reserves for alleviating Cd stress by stimulating the synthesis of glycolysis reaction substances. In addition to the glycolytic pathway, the TCA cycle is the core of cellular energy production, and it is linked with oxidative stress and the carbon balance of plants under different heavy metal conditions [27]. The results revealed that the expression level of carbohydrates such as citric acid, cis-aconitic acid and malic acid in the TCA cycle was upregulated with increasing Cd concentration, suggesting that Cd stress promoted the TCA cycle pathway. Under Cd stress, a series of stress reactions occur in plants, including the production of signal molecules, ROS, and some proteins and metabolites related to Cd resistance [5, 6]. These reactions need to consume large amounts of energy; thus, the upregulation of these metabolites can accelerate the TCA cycle and release more energy to compensate for the energy consumed by the resistance of *S. nigrum* cope with Cd toxicity.

Simultaneously, these carbohydrates are also organic acids, and their accumulation can sequester more Cd in cells, thereby reducing the toxic effect of Cd [30, 31]. Recent reports have demonstrated that citric acid and malic acid can immobilize Cd^2+^ and transport it to vacuoles for compartmentalized isolation [77]. Therefore, *S. nigrum* provides more energy for its detoxification by increasing the accumulation of carbohydrate metabolites and enhancing the TCA cycle, glycolysis and other sugar metabolism pathways, which is a vital response mechanism for its resistance to Cd stress. Based on metabolic data, the amino acid and carbohydrate metabolic pathways, including glutathione metabolism, glyoxylate and dicarboxylate metabolism, TCA and pyruvate metabolism, mainly involved in these DEMs are clearly overviewed in *S. nigrum* roots under five different Cd concentration exposures (Fig. 8c).

#### Conjoint analysis of transcriptomic and metabolomic data

The integrated analyses of transcriptomic and metabolomic results were implemented to further exploit the relationship between DEGs and DEMs in *S. nigrum* roots responsive to Cd stress. First, the DEMs and DEGs were chosen from the results obtained above [31]. The expression of DEMs did not completely show the same trend as related transcripts (Fig. S4). This might be due to the multitudinous regulatory factors during the translation and expression of genes to metabolites, which are related to a sophisticated series of processes [31]. Then, the corresponding transcripts of related enzymes were procured on the basis of metabolism information in the KEGG database [31]. Subsequently, the DEMs and DEGs were apportioned to related metabolic pathways. According to the results, we chose important common pathways of DEMs and DEGs, such as the “TCA cycle”, “glutathione metabolic pathway” and “glyoxylate and dicarboxylate metabolism”, indicating that these three pathways play core roles in the resistance of *S. nigrum* under Cd treatments (Fig. S5-S16). These results partly align with the study by Chen et al. [31].

Based on these maps, we can further obtain the association between DEGs and DEMs that might be involved in these key metabolism pathways in the roots of *S. nigrum* at the molecular level under different Cd concentrations. Some recent researches and our previous results have demonstrated the importance of these three key metabolism pathways in arousing the plant defense system to resist Cd stress [78]. For example, Jiang et al. [67] reported that the upregulated expression of genes encoding proteins associated with the TCA cycle in *Dendrobium officinale* could effectively help to enhance its ability to resist Cd stress. Yue et al. [78] demonstrated that some DEGs related to glutathione metabolism were obviously enriched in GO terms and KEGG pathways, which could contribute to alleviating Cu toxicity in wheat. Similarly, numerous genes encoding proteins related to glyoxylate and dicarboxylate metabolism were upregulated to increase the accumulation of polysaccharides in the rice root cell wall, thereby promoting the vanadium detoxication [79]. Meanwhile, some important enzymes related to these three pathways, including glutathione synthase (EC:6.3.2.3), aminopeptidase N (EC:3.4.11.2), citrate synthase (EC:2.3.3.1), aconitate hydratase (EC:4.2.1.3), isocitrate dehydrogenase (EC:1.1.1.42), malate dehydrogenase (EC:1.1.1.37), and formate dehydrogenase (EC:1.17.1.9), etc., were activated under differernt Cd treatments (Fig. S5-S16). Overall, combined transcriptome and metabolome analyses revealed that *S. nigrum* roots could boost their capacity to withstand Cd stress through adjusting the expression levels of amino acids, carbohydrates and other correlative genes and metabolites. Additionally, more studies will be needed to elucidate the detailed molecular regulatory mechanisms of how the *S. nigrum* root regulates gene and metabolite expression adapt to Cd conditions.

## Conclusion

Our physiological assays demonstrated that Cd stress inhibited growth, increased the accumulation and TF of Cd, disrupted the imbalance of mineral nutrient metabolism, affected photosynthetic pigments, induced the accumulation of H_2_O_2_, MDA, and PRO, and activated antioxidant enzyme defense systems in *S. nigrum*, and these responses were dose/concentration dependent. Many critical DEGs related to mineral nutrients and Cd transport or detoxification, and the antioxidant system were identified, and their expression levels were significantly induced in *S. nigrum* roots with increasing Cd concentrations. In addition, the DEMs analyses demonstrated that Cd stress regulated the variations in numerous amino acids, carbohydrates, lipids, etc. Moreover, we created a modulatory network and obtained several key DEMs related to energy metabolic pathways (e.g., glycolysis, and the TCA cycle) at the metabolic level. Furthermore, based on the integrated transcriptomic and metabolomic analysis, the TCA cycle, glutathione metabolic pathway and glyoxylate and dicarboxylate metabolism were identified as crucial pathways in *S. nigrum* roots, which contribute to building antioxidant enzymes system, Cd sequestration and detoxification. Collectively, these comprehensive physiology, transcriptome, and metabolome analyses not only provide foundational knowledge to investigate the molecular mechanism of Cd detoxification and tolerance in hyperaccumulators, but also offer new insights for improving phytoremediation efficiency using genetic improvement. However, further investigations are needed to elucidate whether the screened DEGs and DEMs are necessary for adaptation to Cd stress in other hyperaccumulators.

### Electronic supplementary material

Below is the link to the electronic supplementary material.

Supplementary Material 1

Supplementary Material 2

Supplementary Material 3

## Data Availability

All data generated or analyzed during this study are included in the manuscript and its Additional files. RNA-Seq data were deposited in NCBI database with SRA accession number PRJNA1049911(https://www.ncbi.nlm.nih.gov/sra/PRJNA1049911).
