# Supplementary material for: Conjoint analysis of physio-biochemical, transcriptomic, and metabolomic reveals the response characteristics of solanum nigrum L. to cadmium stress
Source: BMC Plant Biol. 2024 Jun 17;24:567. doi: 10.1186/s12870-024-05278-z (PMC11181532; doi:10.1186/s12870-024-05278-z)
Supplement: Supplementary file 2 — Supplementary Material 2 [file 12870_2024_5278_MOESM2_ESM.docx]

**Supporting Information**

**for**

**Conjoint analysis of physio-biochemical, transcriptomic, and metabolomic reveals the response characteristics of *Solanum nigrum* L. to cadmium stress**

**Figures: S1-S16**


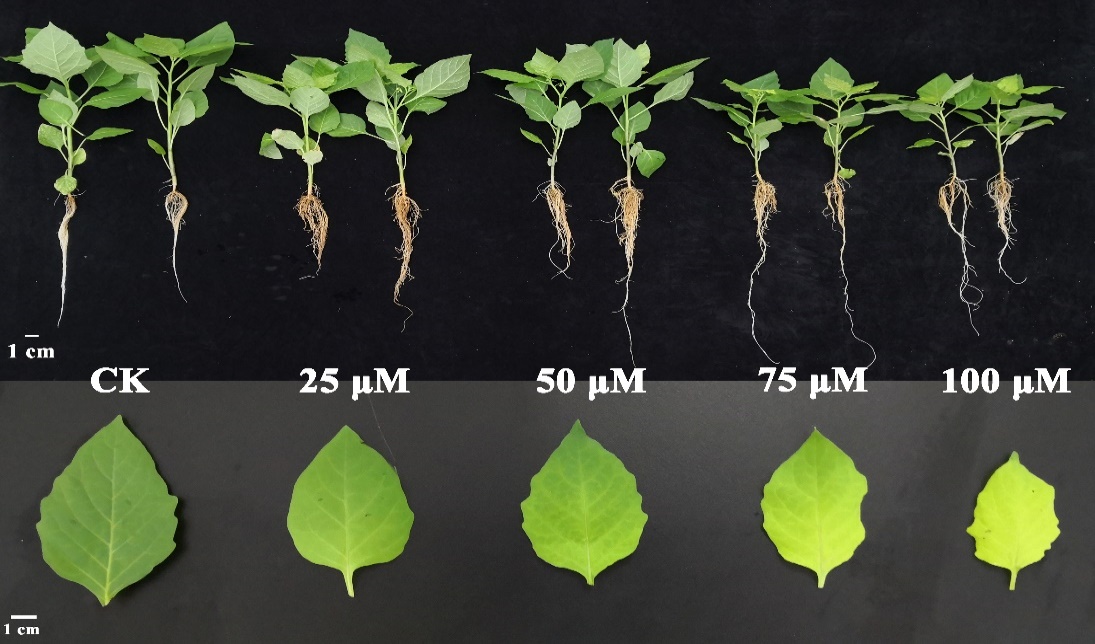


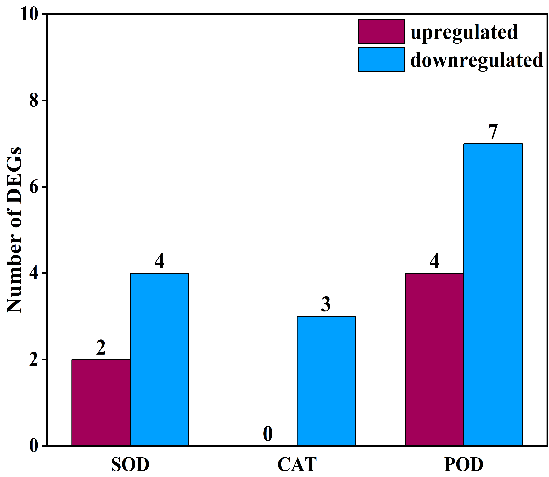
Fig. S1. The growth and leaves (the same location) of *S. nigrum* treated with different Cd concentrations.

1.
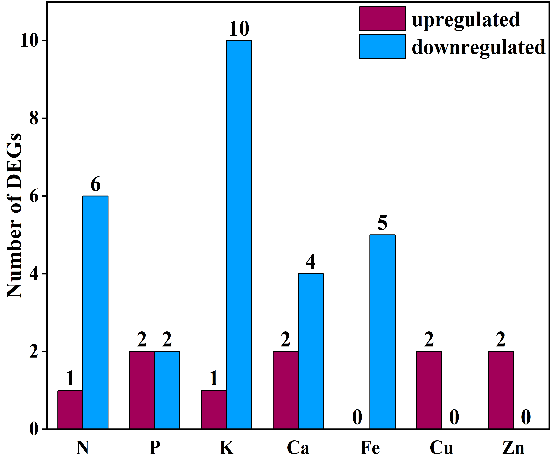
 (b)

Fig. S2. The number of DEGs involved in mineral elements transport (a) and antioxidant enzymes system (b) in S. nigrum root.

Note: Annotated information of related DEGs was presented in Table S2 and Table S5.


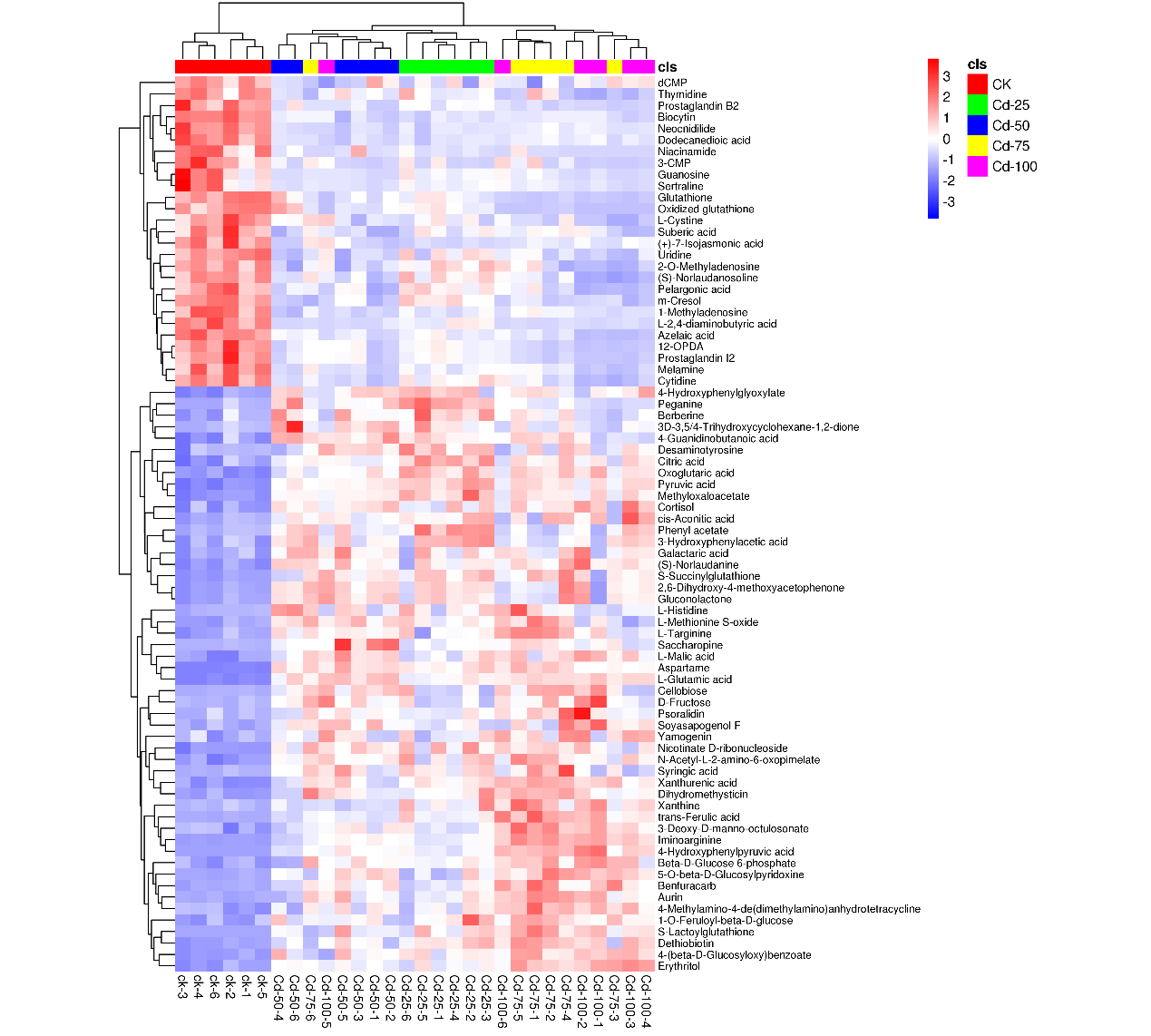


Fig. S3. Heatmap analysis of DEMs in *S. nigrum* roots exposed to different Cd concentrations.

Note: The color scale indicated the relative levels of metabolites in roots, red indicates upregulated metabolites, while blue indicates downregulated metabolites.


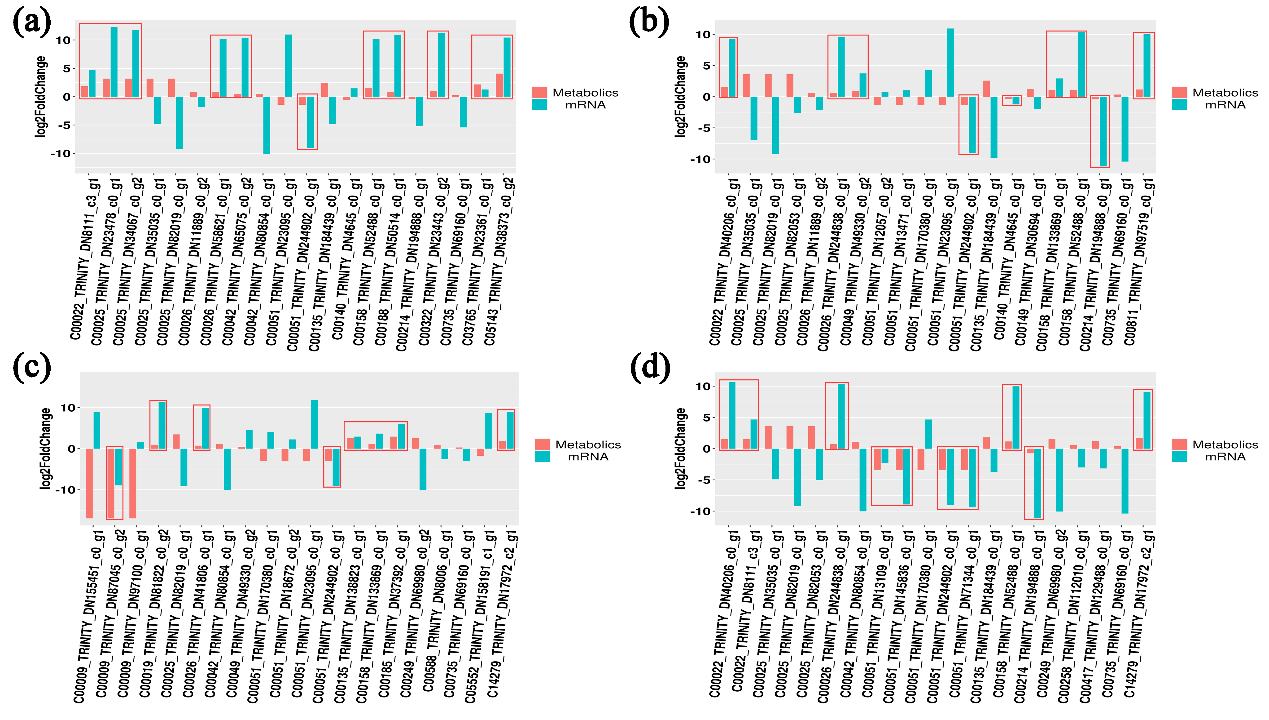


Fig. S4. Expression of DEMs and related transcripts in 25 µM (a), 50 µM (b), 75 µM (c) and 100 µM (d) Cd concentrations stress.

Note: The red box indicates that the expression of DEMs is consistent with the transcripts.


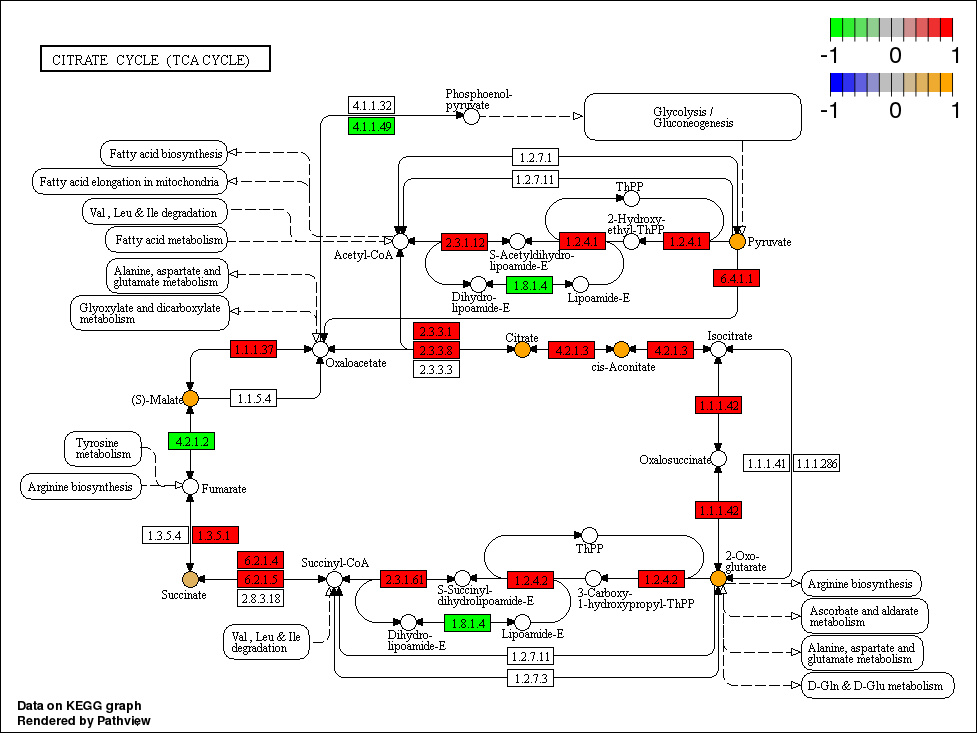


Fig. S5. Visualization results of TCA circulation pathways. (Cd 25 VS CK)

Note: In the figure, the circular nodes are metabolites and the square nodes are enzymes corresponding to the transcript. The differential expression multiples of metabolites from low to high are shown in blue to yellow, and the differential expression multiples of transcripts from low to high are shown in green to red.


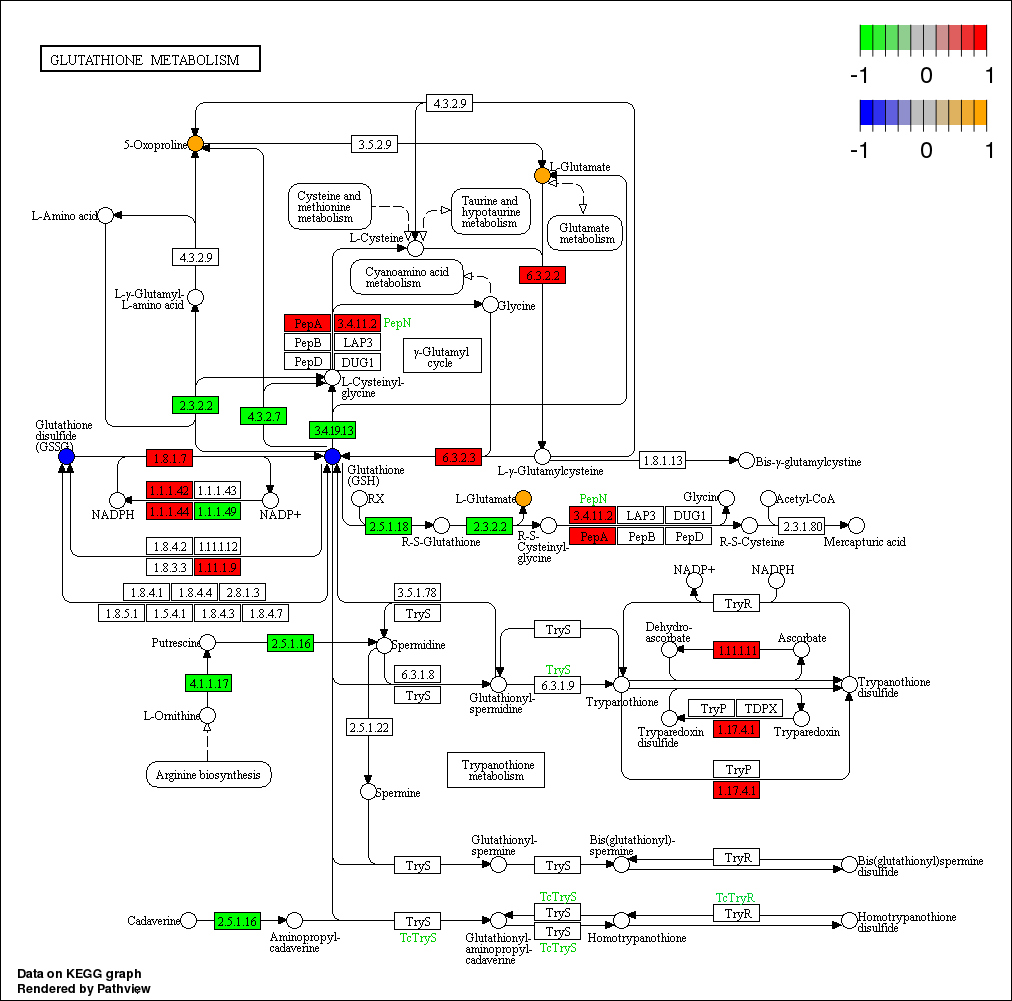


Fig. S6. Visualization results of glutathione metabolic pathway. (Cd 25 VS CK)

Note: In the figure, the circular nodes are metabolites and the square nodes are enzymes corresponding to the transcript. The differential expression multiples of metabolites from low to high are shown in blue to yellow, and the differential expression multiples of transcripts from low to high are shown in green to red.


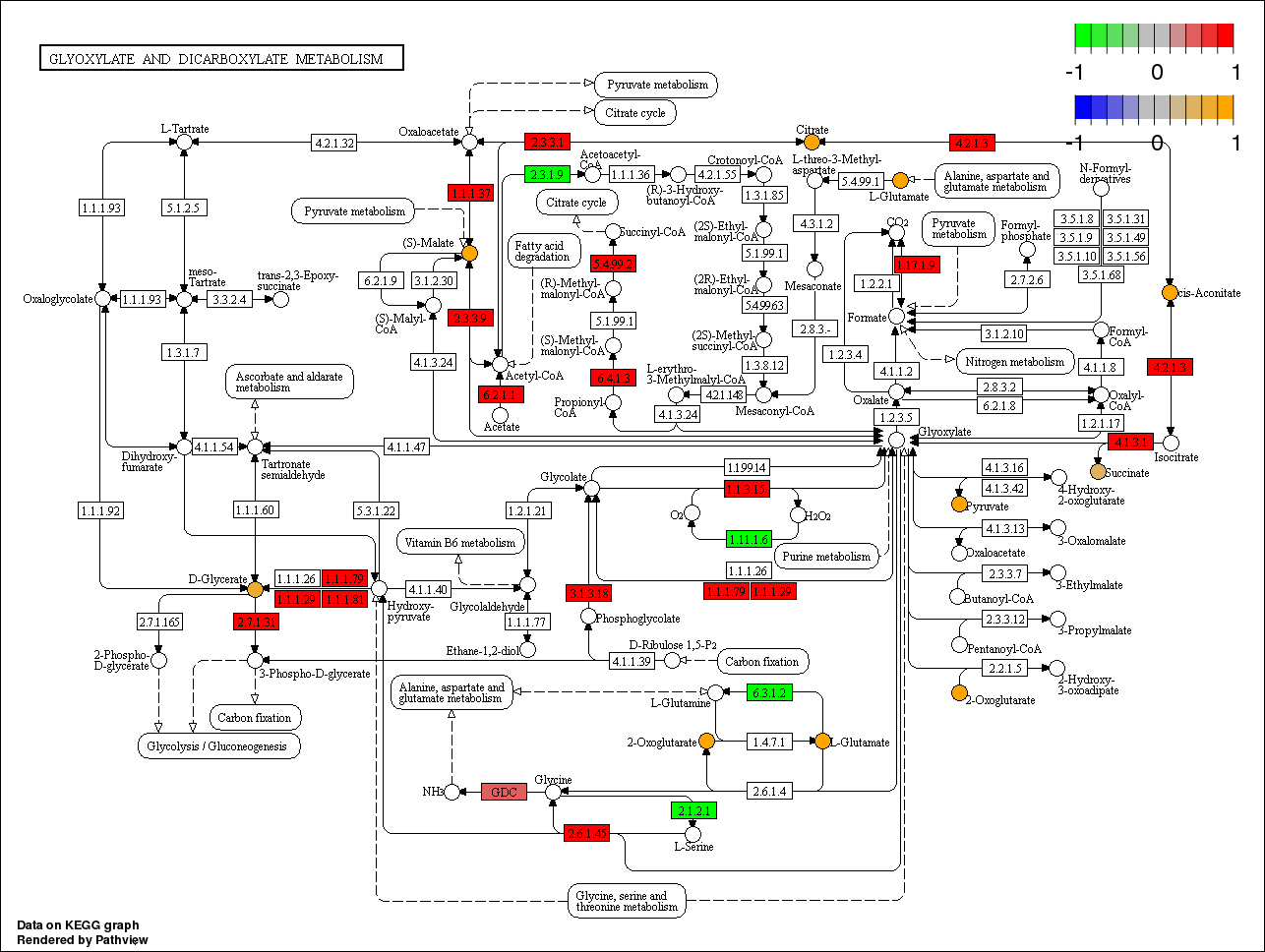


Fig. S7. Visualization results of glyoxylate and dicarboxylate metabolism. (Cd 25 VS CK)

Note: In the figure, the circular nodes are metabolites and the square nodes are enzymes corresponding to the transcript. The differential expression multiples of metabolites from low to high are shown in blue to yellow, and the differential expression multiples of transcripts from low to high are shown in green to red.


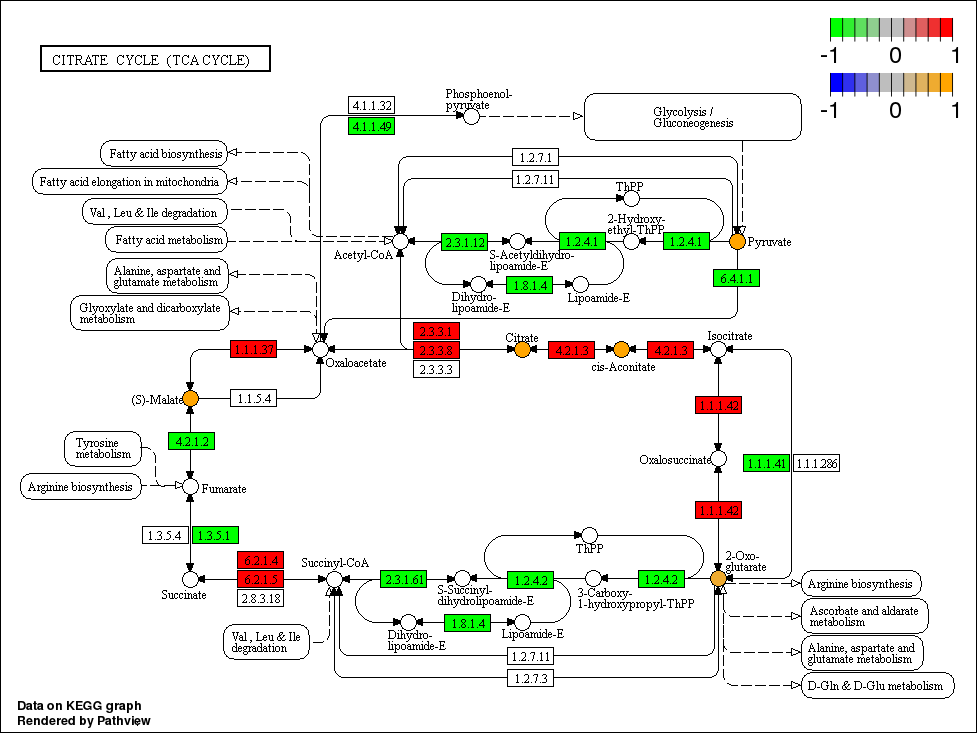


Fig. S8. Visualization results of TCA circulation pathways. (Cd 50 VS CK)

Note: In the figure, the circular nodes are metabolites and the square nodes are enzymes corresponding to the transcript. The differential expression multiples of metabolites from low to high are shown in blue to yellow, and the differential expression multiples of transcripts from low to high are shown in green to red.

**
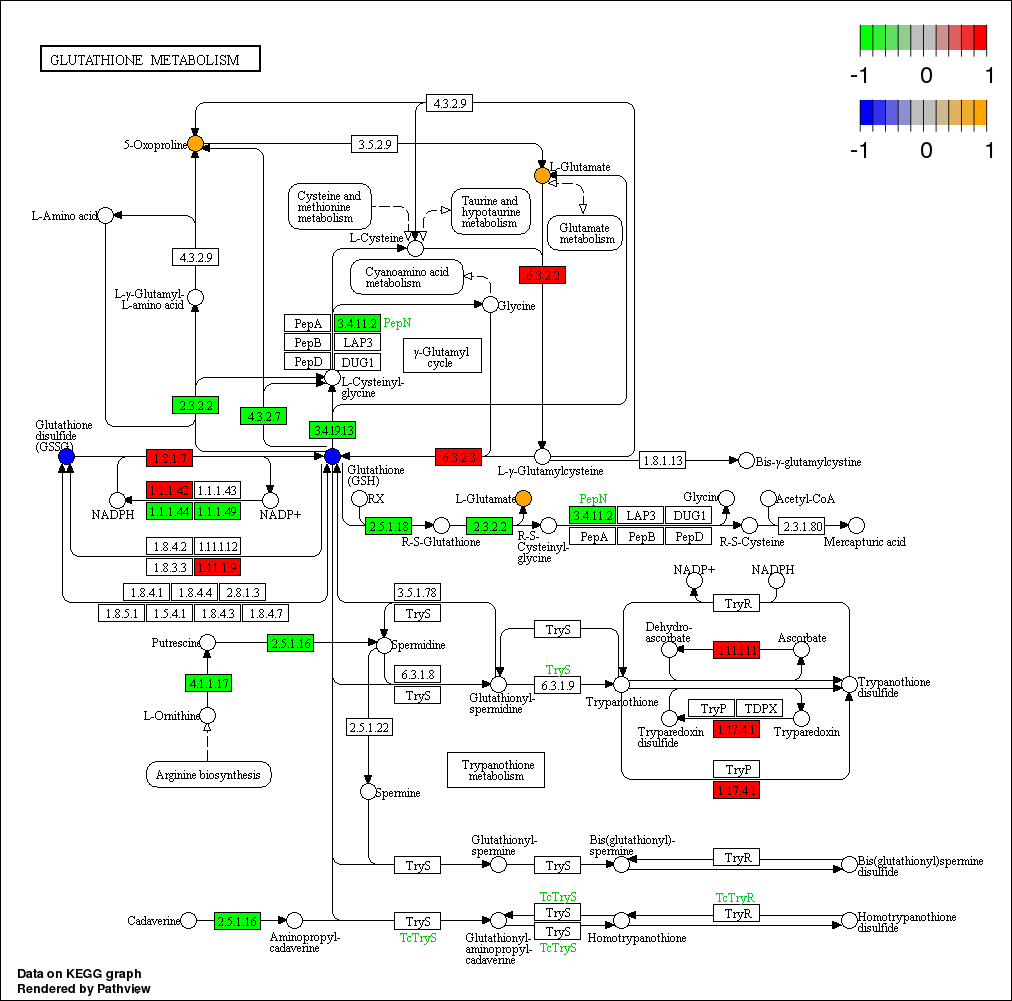
**

Fig. S9. Visualization results of glutathione metabolic pathway. (Cd 50 VS CK)

Note: In the figure, the circular nodes are metabolites and the square nodes are enzymes corresponding to the transcript. The differential expression multiples of metabolites from low to high are shown in blue to yellow, and the differential expression multiples of transcripts from low to high are shown in green to red.


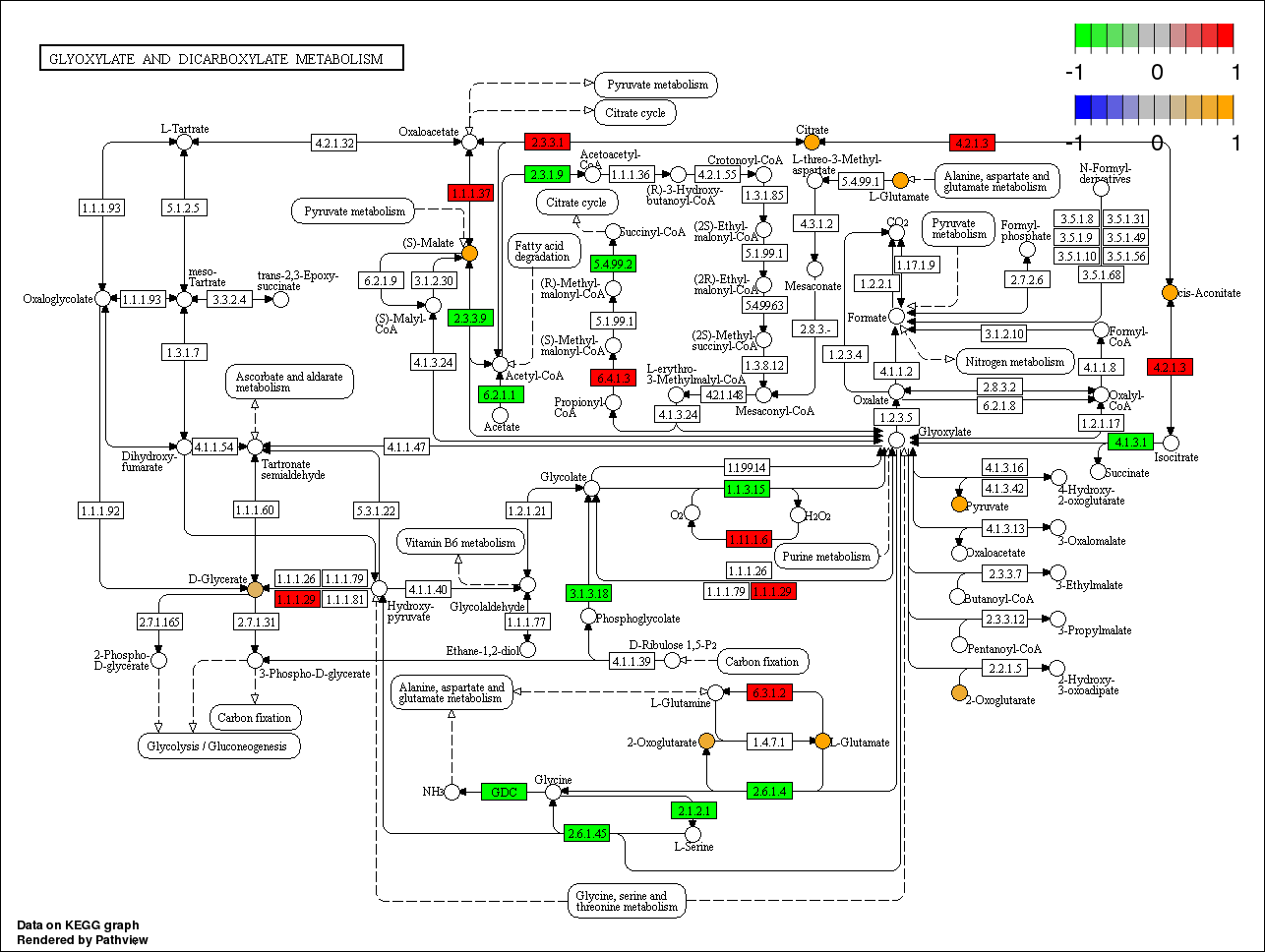


Fig. S10. Visualization results of glyoxylate and dicarboxylate metabolism. (Cd 50 VS CK)

Note: In the figure, the circular nodes are metabolites and the square nodes are enzymes corresponding to the transcript. The differential expression multiples of metabolites from low to high are shown in blue to yellow, and the differential expression multiples of transcripts from low to high are shown in green to red.


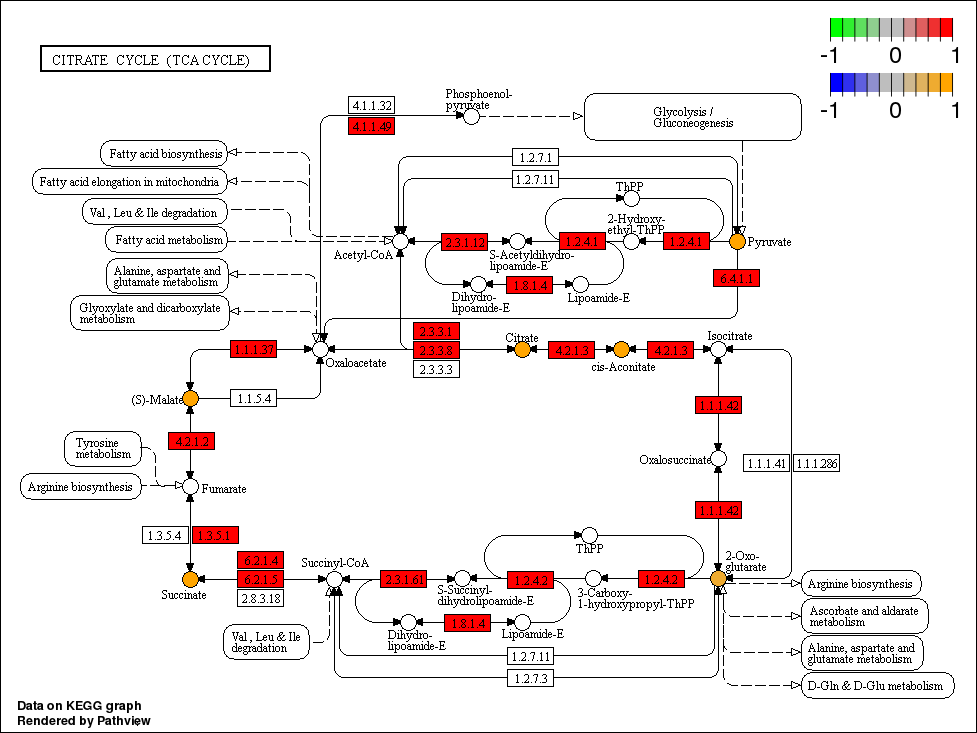


Fig. S11. Visualization results of TCA circulation pathways. (Cd 75 VS CK)

Note: In the figure, the circular nodes are metabolites and the square nodes are enzymes corresponding to the transcript. The differential expression multiples of metabolites from low to high are shown in blue to yellow, and the differential expression multiples of transcripts from low to high are shown in green to red.


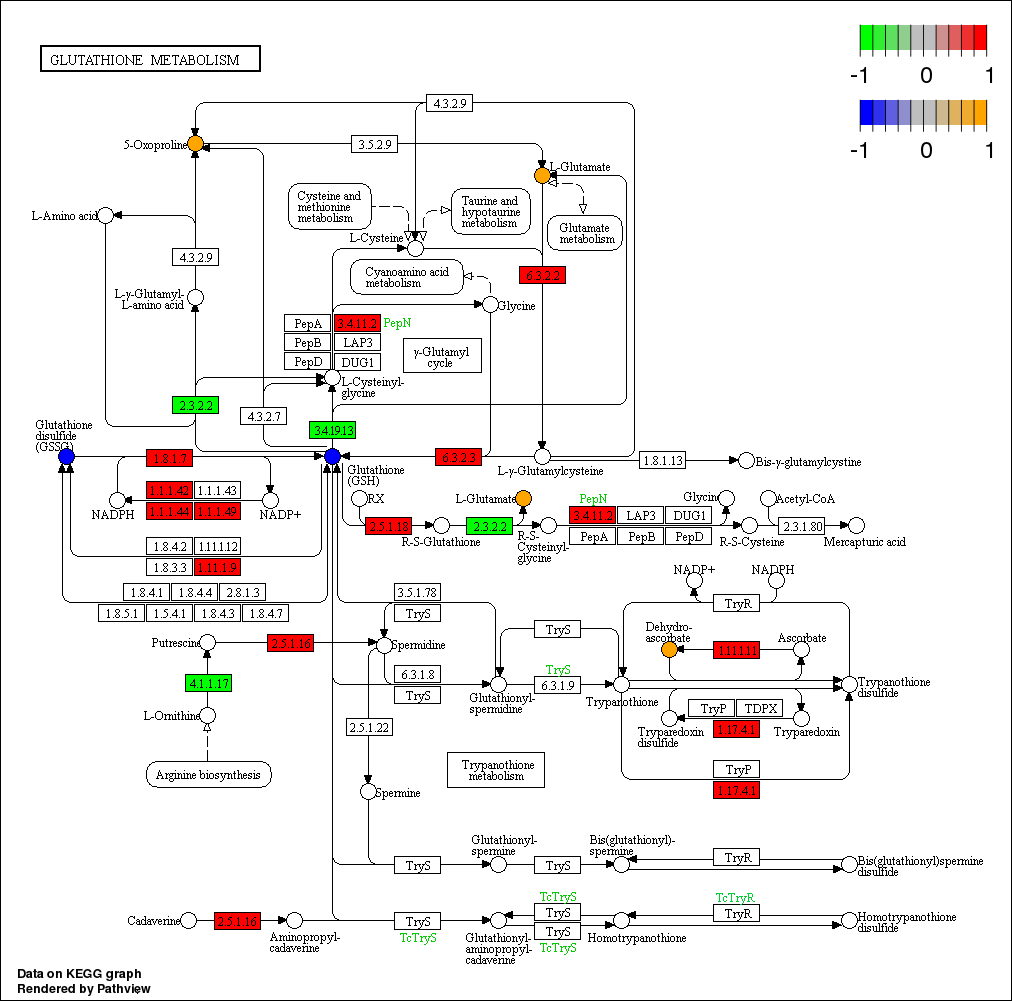


Fig. S12. Visualization results of glutathione metabolic pathway. (Cd 75 VS CK)

Note: In the figure, the circular nodes are metabolites and the square nodes are enzymes corresponding to the transcript. The differential expression multiples of metabolites from low to high are shown in blue to yellow, and the differential expression multiples of transcripts from low to high are shown in green to red.


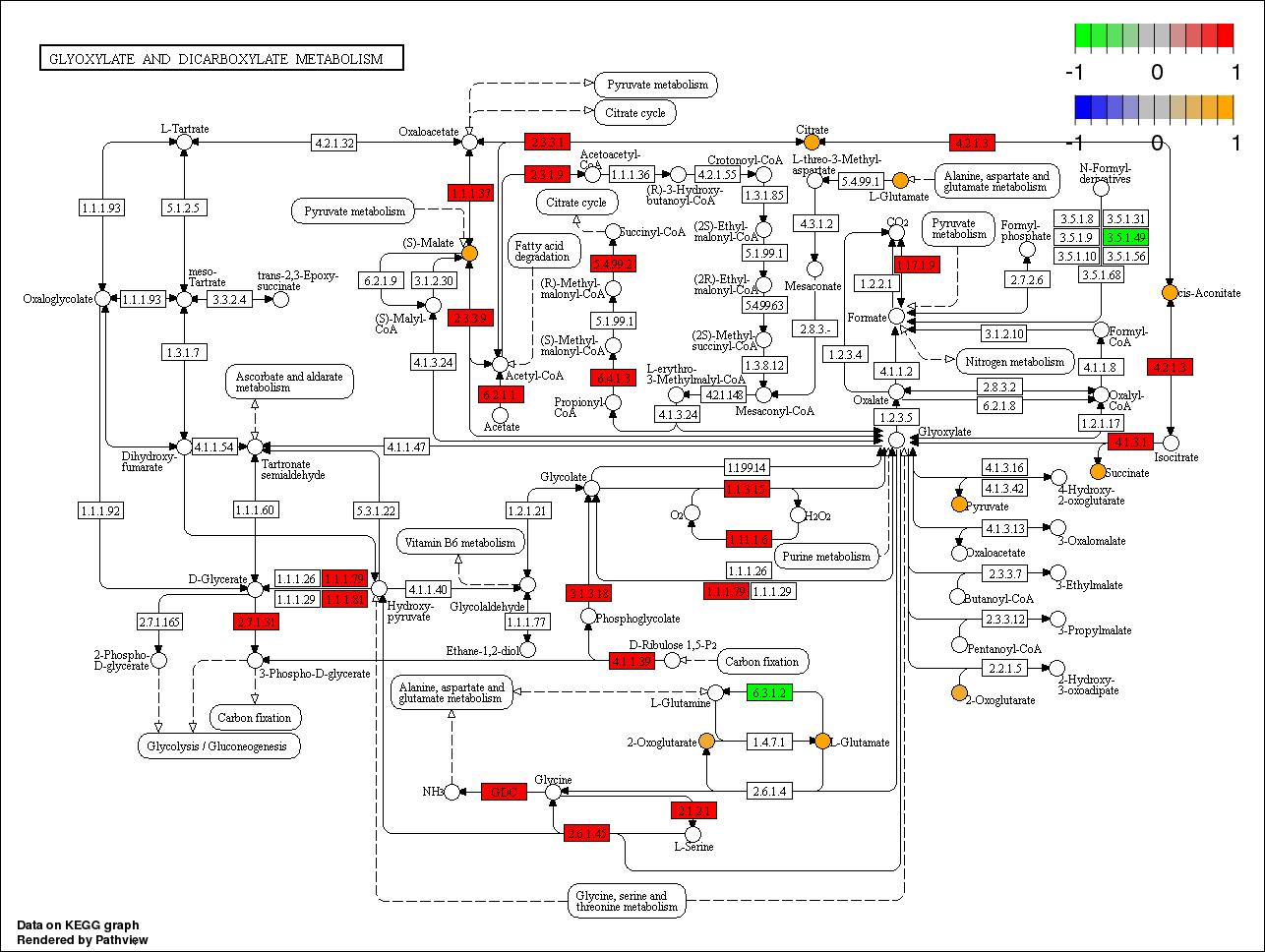


Fig. S13. Visualization results of glyoxylate and dicarboxylate metabolism. (Cd 75 VS CK)

Note: In the figure, the circular nodes are metabolites and the square nodes are enzymes corresponding to the transcript. The differential expression multiples of metabolites from low to high are shown in blue to yellow, and the differential expression multiples of transcripts from low to high are shown in green to red.


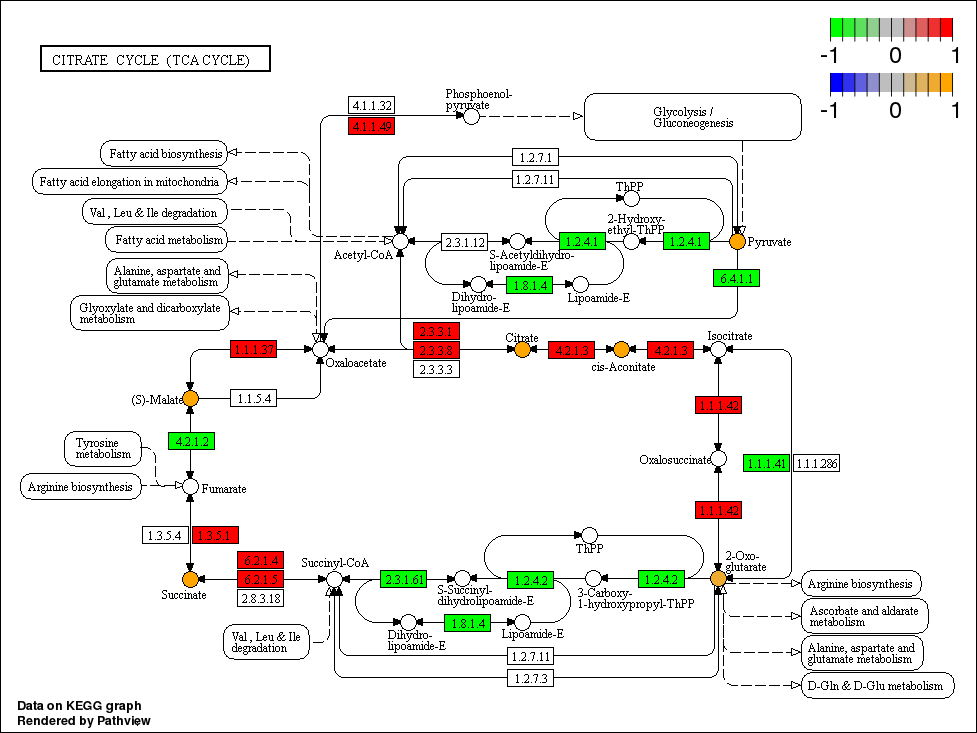


Fig. S14. Visualization results of TCA circulation pathways. (Cd 100 VS CK)

Note: In the figure, the circular nodes are metabolites and the square nodes are enzymes corresponding to the transcript. The differential expression multiples of metabolites from low to high are shown in blue to yellow, and the differential expression multiples of transcripts from low to high are shown in green to red.


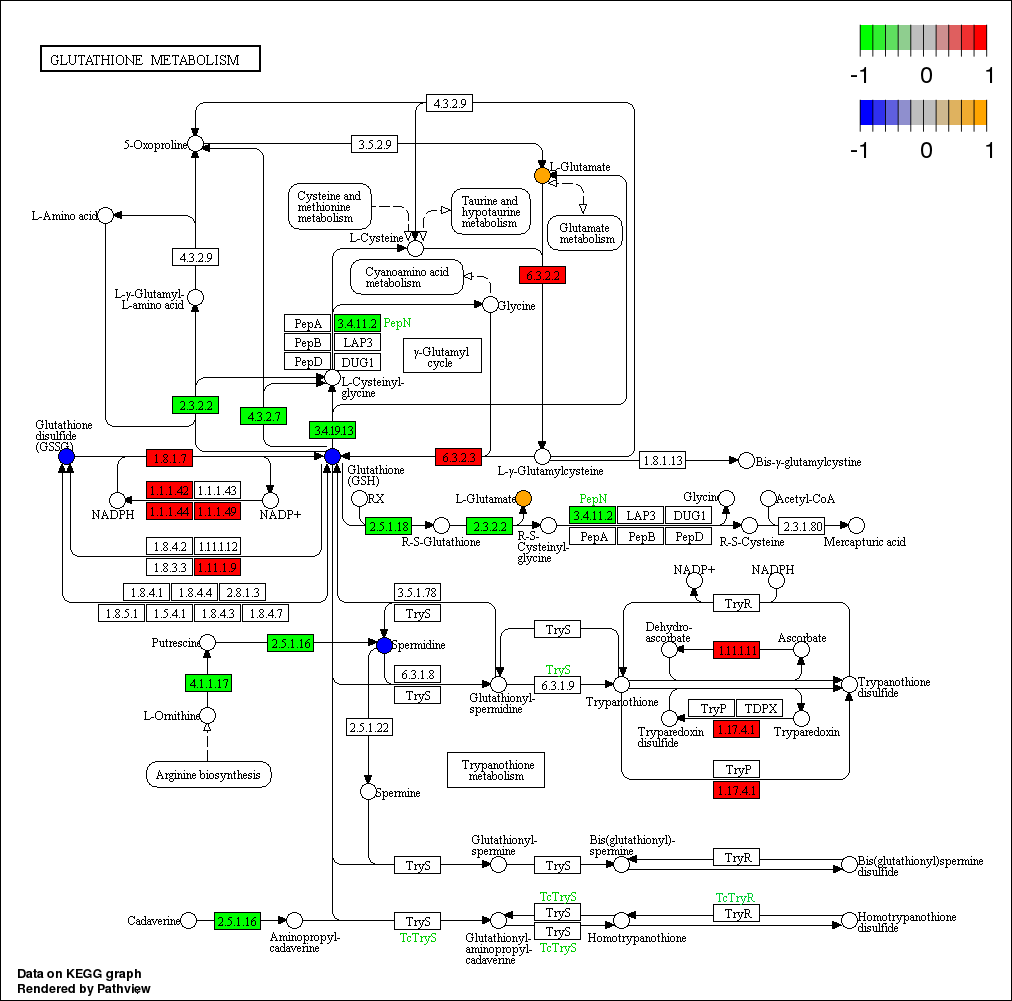


Fig. S15. Visualization results of glutathione metabolic pathway. (Cd 100 VS CK)

Note: In the figure, the circular nodes are metabolites and the square nodes are enzymes corresponding to the transcript. The differential expression multiples of metabolites from low to high are shown in blue to yellow, and the differential expression multiples of transcripts from low to high are shown in green to red.


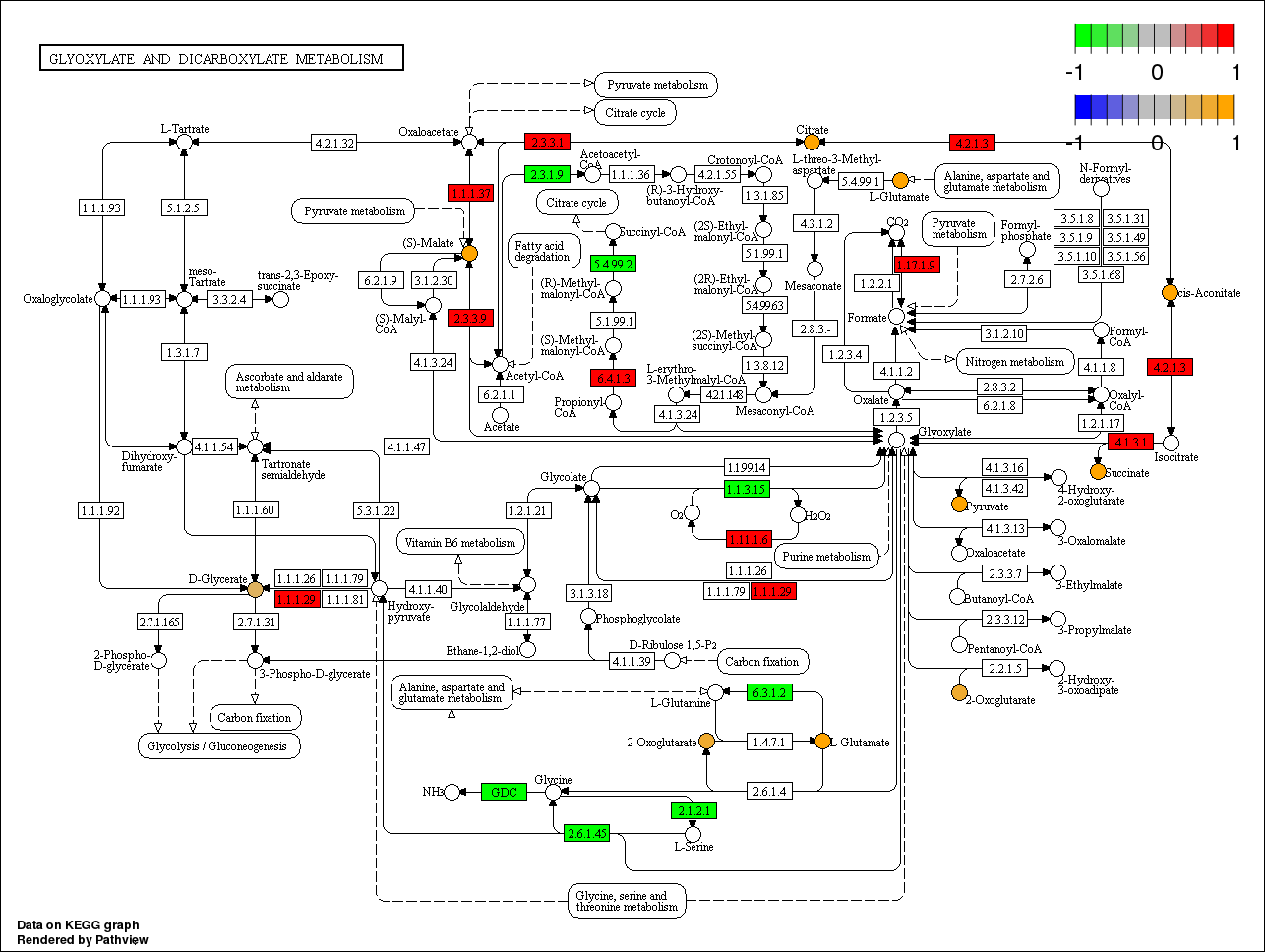


Fig. S16. Visualization results of glyoxylate and dicarboxylate metabolism. (Cd 100 VS CK)

Note: In the figure, the circular nodes are metabolites and the square nodes are enzymes corresponding to the transcript. The differential expression multiples of metabolites from low to high are shown in blue to yellow, and the differential expression multiples of transcripts from low to high are shown in green to red.

**References**

(1) Wang, J.; Chen, X.; Chu, S.; You, Y.; Chi, Y.; Wang, R.; Yang, X.; Hayat, K.; Zhang, D.; Zhou, P. Comparative cytology combined with transcriptomic and metabolomic analyses of Solanum nigrum L. in response to Cd toxicity. *Journal of Hazardous Materials* **2022**, *423*, 127168.

(2) Chen, X.; Wang, J.; You, Y.; Wang, R.; Chu, S.; Chi, Y.; Hayat, K.; Hui, N.; Liu, X.; Zhang, D. When nanoparticle and microbes meet: The effect of multi-walled carbon nanotubes on microbial community and nutrient cycling in hyperaccumulator system. *Journal of Hazardous Materials* **2022**, *423*, 126947.

(3) Chen, X., Wang, J., Wang, R., Zhang, D., Chu, S., Yang, X., Hayat, K., Fan, Z., Cao, X., Ok, Y.S., Zhou, P.. Insights into growth-promoting effect of nanomaterials: Using transcriptomics and metabolomics to reveal the molecular mechanisms of MWCNTs in enhancing hyperaccumulator under heavy metal(loid)s stress. *Journal of Hazardous Materials* **2022**,439, 129640.
