# Supplementary material for: Conjoint analysis of physio-biochemical, transcriptomic, and metabolomic reveals the response characteristics of solanum nigrum L. to cadmium stress
Source: BMC Plant Biol. 2024 Jun 17;24:567. doi: 10.1186/s12870-024-05278-z (PMC11181532; doi:10.1186/s12870-024-05278-z)
Supplement: Supplementary file 3 — Supplementary Material 3 [file 12870_2024_5278_MOESM3_ESM.docx]

**Text S1 Metabolomic analysis**

**Extraction of metabolite.** Weight 200 mg (± 1%) of the *S. nigrum* roots sample in a 2 ml EP tube, and add 0.6 ml 2-chlorophenylalanine (4 ppm) methanol (-20 ℃), vortex for 30 seconds. Then add 100 mg glass beads, put them into the tissue grinder, and grind for 90 s at 55 Hz. After ultrasound for 15 min at room temperature, the root sample was centrifugation at 12000 rpm at 4 ℃ for 10 min, took 200 μL supernatant and filtered through 0.22 μm membrane, and added the filtrate into the detection bottle. Take 20 μL from each sample to the quality control (QC) samples; (These QC samples were used to monitor deviations of the analytical results from these pool mixtures and compare them to the errors caused by the analytical instrument itself). Subsequently, use the samples for LC-MS detection at Personal Biotechnology Co., Ltd. (Shanghai, China).

**Analysis conditions.** The metabolomics analysis of *S. nigrum* roots was performed with the following chromatographic condition: Chromatographic separation was accomplished in an Thermo Ultimate 3000 system equipped with an ACQUITY UPLC® HSS T3 (150×2.1 mm, 1.8 μm, Waters) column maintained at 40 ℃. The temperature of the autosampler was 8 ℃. Gradient elution of analytes was carried out with 0.1% formic acid in water (C) and 0.1% formic acid in acetonitrile (D) or 5 mM ammonium formate in water (A) and acetonitrile (B) at a flow rate of 0.25 mL/min. Injection of 2 μL of each sample was done after equilibration. An increasing linear gradient of solvent B (v/v) was used as follows: 0~1 min, 2% B/D; 1~9 min, 2%~50% B/D; 9~12 min, 50%~98% B/D; 12~13.5 min, 98% B/D; 13.5~14 min, 98%~2% B/D; 14~20 min, 2% D-positive model (14~17 min, 2% B-negative model). And the mass spectrometry conditions were as follows. The ESI-MSn experiments were executed on the Thermo Q Exactive Focus mass spectrometer with the spray voltage of 3.5 kV and -2.5 kV in positive and negative modes, respectively. Sheath gas and auxiliary gas were set at 30 and 10 arbitrary units, respectively. The capillary temperature was 325 ℃. respectively. The Orbitrap analyzer scanned over a mass range of m/z 81-1 000 for full scan at a mass resolution of 70 000. Data-dependent acquisition (DDA) MS/MS experiments were performed with an HCD scan. The normalized collision energy was 30 eV. Dynamic exclusion was implemented to remove some unnecessary information in MS/MS spectra.

**Date analysis.** The unprocessed MS dataset was transformed to the mzML format by ProteoWizard software. Further data processing including peaks identification, peaks filtration, peaks alignment, and internal standard normalization was performed by the XCMS in R package (version 3.3.2). The processed data was imported into SIMCA software (version 14.1) for multivariate analysis, including Principal Component Analysis (PCA) and Orthogonal Projections to Latent Structures Discriminant Analysis (OPLS-DA). The OPLS-DA analysis further served to generate values of the Variable Importance in the Projection (VIP). The differentially expressed metabolites (DEMs) were screened with the VIP > 1 and P-values < 0.05 from Student’s t-test on the normalized peak areas. The KEGG (http://www.genome.jp/kegg/), Human Metabolome Database (HMDB) (http://www.hmdb.ca/), and MetaboAnalyst (http://www.metaboanalyst.ca/) were used for the metabolic pathway analysis.

**Transcriptomic analysis**

Total RNA from *S. nigrum* roots was extracted by pre-chilled TRIzol Reagent according to the instructions provided by the manufacturer (Invitrogen, Carlsbad, USA). Then, the NanDrop 2000 (Thermo Scientific, Waltham, USA) and Agilent Bioanalyzer 2100 System (Agilent Technologies, Santa Clara, USA) were used to detect the quality and integrity of total RNA, respectively. After RNA extraction, purification and library construction, RNA sequencing was performed using Next-Generation Sequencing analysis based on an Illumina HiSeq platform at Personal Biotechnology Co., Ltd. (Shanghai, China). Subsequently, the high-quality clean reads were obtained after removing low-quality reads and adapter reads, and then submitted to Trinity software for the further de novo assembly. Here, the longest transcript was selected as unigene after clustering. For gene functional annotation, the unigenes were annotated on the basis of NCBI non-redundant protein sequences (NR), evolutionary genealogy of genes: Non-supervised Orthologous Groups (eggNOG), Swiss-Prot Protein Sequence (Swiss-Prot), Protein family (Pfam), Kyoto Encyclopedia of Genes and Genome (KEGG), and Gene Ontology (GO). Furthermore, RSEM software was applied to assess the unigenes expression levels. Moreover, the differentially expressed genes (DEGs) between the control and Cd-treated group were identified based on |log2FoldChange|＞ 1 and *p*-value＜ 0.05 by using the DESeq R package (An et al., 2022). Then, topGO R software and KOBAS software were used to identify the enrichment of DEGs in the Go functions and KEGG pathways. More information was described our previously.

Reference:

[1] Yu, G., et al. (2022). "Integrated transcriptome and metabolome analysis reveals the mechanism of tolerance to manganese and cadmium toxicity in the Mn/Cd hyperaccumulator Celosia argentea Linn." J Hazard Mater 443(Pt A): 130206.

[2] Chen, X., et al. (2022). "Insights into growth-promoting effect of nanomaterials: Using transcriptomics and metabolomics to reveal the molecular mechanisms of MWCNTs in enhancing hyperaccumulator under heavy metal(loid)s stress." J Hazard Mater 439: 129640.

[3] Wang, J., et al. (2021). "Comparative cytology combined with transcriptomic and metabolomic analyses of Solanum nigrum L. in response to Cd toxicity." J Hazard Mater 423(Pt B): 127168.

[4] Chen, S., et al. (2021). "Transcriptomics of different tissues of blueberry and diversity analysis of rhizosphere fungi under cadmium stress." Bmc Plant Biology 21(1):389.

[5] Wang, M. Q., et al. (2019). "Transcriptomic analysis of Verbena bonariensis roots in response to cadmium stress." Bmc Genomics 20(1): 877.
